# Efficacy of Intensified Hygiene Measures with or without the Addition of Doxycycline in the Management of Filarial Lymphedema: A Randomized Double-Blind, Placebo-Controlled Clinical Trial in Tanzania

**DOI:** 10.4269/ajtmh.24-0049

**Published:** 2024-08-27

**Authors:** Abdallah Ngenya, Ute Klarmann-Schulz, Winfrida John, Patricia Jebett Korir, Mathias Kamugisha, Jennifer Nadal, Dennis Moshi, Arcangelo Ricchiuto, Ndekya Oriyo, Sarah Mary Sullivan, Ruth Laizer, John Horton, Max Demitrius, Anja Feichtner, Thomas F. Marandu, Yusuph Mgaya, Angelika Kellings, Inge Kroidl, John Ogondiek, Janina M. Kuehlwein, Leonard Masagati, Charles Mackenzie, Maureen Mosoba, Sacha Horn, Kheri Kagya, Samuel Wanji, Wilfred Mandara, Linda Batsa Debrah, Eric A. Ottesen, Alexander Yaw Debrah, Upendo Mwingira, Achim Hoerauf, Akili Kalinga

**Affiliations:** ^1^ National Institute for Medical Research, Dar es Salaam, Tanzania;; ^2^ Institute for Medical Microbiology, Immunology and Parasitology, University Hospital Bonn, Bonn, Germany;; ^3^ German Center for Infection Research, partner site Bonn-Cologne, Bonn, Germany;; ^4^ Institute for Medical Biometry, Informatics and Epidemiology, University Hospital Bonn, Bonn, Germany;; ^5^ Neglected Tropical Diseases Support Center, Task Force for Global Health, Decatur, Georgia;; ^6^ Kilimanjaro Clinical Research Institute, Moshi, Tanzania;; ^7^ Tropical Projects, Hitchin, United Kingdom;; ^8^ Division of Infectious Diseases and Tropical Medicine, Medical Center of the Ludwig-Maximilians-University, Munich, Germany;; ^9^ German Center for Infection Research, partner site Munich, Munich, Germany;; ^10^ University of Dar es Salaam–Mbeya College of Health and Allied Sciences, Mbeya, Tanzania;; ^11^ Clinical Study Core Unit Bonn, Institute of Clinical Chemistry and Clinical Pharmacology, University of Bonn, Bonn, Germany;; ^12^ Center for International Health, Ludwig-Maximilians-University, Munich, Germany;; ^13^ Regional Medical Office, Lindi Municipal Council, Lindi Region, Tanzania;; ^14^ Department of Microbiology and Parasitology, University of Buea, Buea, Cameroon;; ^15^ Kumasi Centre for Collaborative Research in Tropical Medicine, Kwame Nkrumah University of Science and Technology, Kumasi, Ghana;; ^16^ Department of Clinical Microbiology, School of Medicine and Dentistry, Kwame Nkrumah University of Science and Technology, Kumasi, Ghana;; ^17^ German–West African Center for Global Health and Pandemic Prevention, partner site Kumasi, Kumasi, Ghana;; ^18^ Faculty of Allied Health Sciences, Kwame Nkrumah University of Science and Technology, Kumasi, Ghana;; ^19^ RTI International, Washington, District of Columbia;; ^20^ German–West African Center for Global Health and Pandemic Prevention, partner site Bonn, Bonn, Germany

## Abstract

Lymphedema, hydrocele, and acute adenolymphangitis (ADL) are chronically disabling consequences in patients with lymphatic filariasis (LF). Provision of morbidity management and disability prevention and concurrent mass drug administration of anthelmintics are two pillars for elimination of LF. This study assessed the impact of strict hygiene protocols with or without doxycycline on the progression of filarial lymphedema. A randomized, placebo-controlled, double-blind trial was conducted in two regions in Tanzania. We enrolled 362 participants with lymphedema stages 1–3 assigned into three treatment groups of doxycycline 200 mg once daily, doxycycline 100 mg once daily, or matching placebo for 42 days in addition to hygiene measures. The participants were followed every 2 months for 2 years. Twenty-four months after treatment onset, 17.7% of participants displayed improved limb conditions, including 15/104 (14.4%) in the doxycycline 200 mg group, 16/105 (15.2%) in the doxycycline 100 mg group, and 25/107 (23.4%) in the placebo group. During the first 6 months after treatment, the number of participants experiencing an ADL attack was significantly lower in the doxycycline groups than in the placebo group. The study also found that hygiene was one of the factors associated with preventing the occurrence of acute attacks over the whole study period. Doxycycline 100 mg was a significant factor for the halt of progression (odds ratio: 0.53, *P* = 0.0239) when both legs if affected at baseline were considered. These findings emphasize the importance of practicing hygiene in reducing the occurrence of ADL attacks and the benefits of doxycycline with regards to acute attacks and halt of progression.

## INTRODUCTION

Lymphatic filariasis (LF) is an incapacitating infectious neglected tropical disease (NTD) caused by *Wuchereria bancrofti*, *Brugia malayi*, or *Brugia timori* parasites.^1^^,^^2^ Currently, 51.4 million people are estimated to be impacted by LF globally,^1^ with 40 million people reported to have LF-related morbidities: 25 million are men living with hydrocele, and 15 million are living with lymphedema (LE).^2^^,^^3^

Upper and lower limb LE, hydrocele, and acute episodes of adenolymphangitis (ADL) are common LF-related morbidities that are not only painful and disfiguring but also have been linked to a low quality of life (QoL). Breast, genital, and upper limb LE are less frequently observed clinical manifestations.^3^ Lymphatic filariasis-related LE is of progressive onset and may be accompanied by skin alterations such as thickening of the skin and formation of knobs, folds, and mossy lesions.^4^ The WHO Road Map for NTDs (2021–2030) is committed to eliminating LF as a public health problem and aligns with the United Nations Sustainable Development Goal of good health and well-being.^5^^,^^6^ The main interventions used to eliminate LF are administration of preventive chemotherapy using albendazole combined with ivermectin and/or diethylcarbamazine in mass drug administration (MDA) to interrupt transmission of the infection and morbidity management and disability prevention (MMDP) services specifically for those affected with LE and/or hydrocele.^7^^–^^9^ According to the latest report from the Ministry of Health, Tanzania has made significant progress in reducing the prevalence and intensity of NTDs in the past decade.^9^

So far, large surveys of LF morbidities have been performed in the six districts of the Dar es Salaam region in 2017^10^ and in one district of the Lindi region in 2020,^11^ where 6,000 patients with hydrocele and 1,904 patients with LE were identified, respectively. Therefore, from these surveys and other methods of tracking cases, nearly 10,740 surgeries of hydrocele and hernia have been conducted between September 2008 and October 2023 through funding from different partners, including UKAID, CROWN Agents, END FUND, Centre for Neglected Tropical Diseases, and EQUINOR, etc. On the other hand, the provision of MMDP services for LE patients has been limited to a few areas of Tanzania, including the ancient Samaritan Lymphedema Clinic at the National Institute for Medical Research in Dar es Salaam. As in 2023, about 5,500 LE patients have been trained on the minimum essential package of care (Ministry of Health, unpublished report).^11^^,^^12^

The management of LE relies principally on nonpharmaceutical approaches (hygiene-based treatment, decongestive lymphatic therapy, decompression, bandaging, and physiotherapy.^13^ A combination of nonpharmaceutical and pharmacologic treatment with nonsteroidal anti-inflammatory drugs and selenium is also practiced but to a lesser extent.^14^^,^^15^ Hygiene-based management of LE focuses on cleaning the affected limbs, applying appropriate topical antibiotics and antifungals, exercising, elevating the limb, inspection for entry lesions, and wearing proper footwear to prevent infection that could lead to ADL attacks and result in severe, debilitating LE.^16^ In addition, hygiene-based treatment has been demonstrated to be helpful in slowing the progression of LE; however, in resource-limited settings, availability of essential materials necessary for limb care is a challenge. Furthermore, strict adherence to the prescribed procedures is difficult for the patients.^17^

Despite the global understanding of hygiene as a key component in managing LE, only a limited number of individuals affected by this condition have access to such care.^7^

Having a drug that targets the endosymbiotic bacterium *Wolbachia* (essential for the survival and reproduction of adult filarial worms) and that is macrofilaricidal, especially with a concurrent reduction of inflammatory angiopoietic factors, could significantly diminish the parasite reservoir and advance toward its ultimate elimination.^18^ Proof of concept for the use of doxycycline (C) 200 mg/day for 6 weeks as a female worm-sterilizing or “macrofilaricidal” therapy has been demonstrated in an extensive series of field trials.^19^^–^^25^ Additionally, the efficacy of DOX 200 mg for 6 weeks for selective treatment of LE by reversing or stopping its progression in patients with stages 1–3, irrespective of active filarial infection, has been shown.^26^ Because there was a need to conduct similar studies in different epidemiological settings in other countries and with a larger sample size than in the earlier DOX trial in Ghana^26^ and elsewhere,^27^^–^^29^ the current study was designed to be conducted in Tanzania.

The goal of the current study was to determine whether a 6-week course of DOX treatment, in addition to the essential package of care recommended by the WHO, would arrest or reverse the worsening of filarial LE among study participants in the Lindi and Pwani regions in Tanzania.

## MATERIALS AND METHODS

### Study sites.

The study was conducted in the Lindi and Pwani regions of Tanzania. Lindi is a coastal town located along the Indian Ocean in southeastern Tanzania. According to the 2022 population and housing census, the population of the region is 1,194,028.^30^ The region has a total of six district councils where LF is endemic (Supplemental Figure 1).^30^

The initial LF mapping in Lindi in 2001 using the immunochromatographic test (ICT) indicated an overall prevalence of 51.8% (National NTD Control Program, unpublished report). The MDA started in 2002. After 11 rounds, in 2015–2016, a sentinel site assessment survey, which covered all six districts of Lindi region, namely Lindi Urban, Lindi Rural, Ruangwa, Nachingwea, Liwale, and Kilwa, showed an LF prevalence of 4.7% by use of the filariasis test strip (FTS) (National NTD Control Program, unpublished data). The current randomized clinical trial (RCT) was conducted in Lindi in 19 (13.7%) urban, 17 (12.2%) semiurban, and 103 (74.1%) rural communities.

The Pwani region lies on the eastern part of Tanzania’s mainland. According to “The 2022 Population and Housing Census,” the region’s population is 2,024,947.^30^ The initial LF mapping in the Pwani region conducted from 1998 to 2001 using the ICT indicated a prevalence of 44.5%. The sentinel site assessment survey done from 2015 to 2017 by use of the FTS showed an overall prevalence in all eight districts, namely Bagamoyo, Chalinze, Kibaha, Kibaha Urban, Kisarawe, Mafia, Mkuranga, and Rufiji, of 2.3% (National NTD Control Program, unpublished data). The current RCT was conducted in five out of seven district councils (Kibaha, Kisarawe, Mlandizi, Chalinze, Kibiti) where LF is endemic (Supplemental Figure 2),^30^ and the surveyed wards were located in 3 (2.7%) semiurban and 107 (97.3%) rural settings.

### Study population eligibility criteria and sampling procedures.

Public announcements were made in the villages to inform individuals with LF morbidities to gather at the nearest health facilities in their respective residence areas. Thereafter, they were given general health education about NTDs, particularly LF and interventions for its elimination as a public health problem. Individuals aged 14–65 years who showed interest in participating received an essential package of care for LE, as defined in the WHO guidelines, and were given further details about the RCT.^4^^,^^7^ All instructions on the use of the essential care package were communicated in Kiswahili, the national language.^4^^,^^7^ All interested individuals were screened for eligibility after signing the informed consent forms.

Inclusion criteria restricted enrollment to individuals with LE in at least one leg (based on the staging described by Dreyer et al.),^31^ aged between 14 and 65 years, and either male or nonpregnant, nonbreastfeeding females.^26^ Women of childbearing potential were required to use effective contraception. Other inclusion criteria included a body weight of ≥40 kg, residency in an area of endemicity for ≥2 years, ability to provide informed consent, and adherence to hygiene practices.

Exclusion criteria were absence of LE or presence of stage 7 LE, age <14 or >65 years, body weight <40 kg, pregnant or breastfeeding, lack of contraception use, hepatic or renal dysfunction, history of adverse reactions to the trial drug, significant medical or psychiatric disorders, photosensitivity reactions, and concurrent use of certain medications. The exclusion criteria based on laboratory values included a hemoglobin level of <8 g/dL, a neutrophil count of <1,100/mm^3^, a platelet count of <100,000/mm^3^, a creatinine level greater than two times the upper limit of normal, an aspartate aminotransferase (glutamic-oxaloacetic transaminase) level greater than two times the upper limit of normal, an alanine aminotransferase (glutamic-pyruvic transaminase) level greater than two times the upper limit of normal, a gamma-glutamyl transferase level greater than two times the upper limit of normal, and a positive urine pregnancy test for women.

### Study design.

This study was a prospective, randomized, placebo-controlled, double-blind, parallel-group interventional phase II trial that included participants with LE stages 1–3 (group A). Four other studies with almost identical protocols and using the same drug supplies were carried out in parallel in Ghana, Mali, Sri Lanka, and India (see the other articles in this series).

Additionally, a smaller number of participants with LE stages 4–6 were included in a prospective, multinational, randomized, placebo-controlled, double-blind, parallel-group interventional pilot trial (group B). However, the current paper is only about the participants from group A. The results of group B will be published separately.

### Interventions and randomization.

Eligible participants for group A were randomized into three different treatment groups: 1) DOX 200 mg/day for 6 weeks (to confirm the results from previous trials),^18^^,^^26^ 2) DOX 100 mg/day for 6 weeks, and 3) placebo matching DOX for 6 weeks.

Every treatment was administered in addition to standardized and intensified hygiene measures. Additionally, the participants were encouraged to take part in the annual MDA during the trial period.

The DOX tablets, consisting of doxycycline hyclate, were produced by Remedica, Limassol, Cyprus. The manufacture of the placebo as well as supply, analysis, blistering, packaging, and distribution was undertaken by Piramal Healthcare Ltd., Morpeth, United Kingdom, a company with strong experience in good manufacturing practice production of clinical trial supplies, as described by Horton et al.^7^

Randomization lists were generated by the manufacturer of the study drugs (Piramal Healthcare Ltd.) by using block randomization. Trial participants, care providers, and outcome assessors were blinded to the trial drugs received by the participants.

### Sample size estimation for group A (LE stages 1–3).

The hypothesis for the sample size calculation was that DOX is superior to placebo. The primary outcome was defined as lack of progression (worsening) of LE (stage reduction or same stage as baseline) at examination 24 months after the onset of treatment. DOX 200 mg/day was first to be tested for superiority to placebo. If the superiority of DOX 200 mg/day were confirmed, DOX 100 mg/day was to be subsequently tested for superiority to placebo. In case both treatment groups rejected their null hypothesis, testing for noninferiority of DOX 200 mg versus DOX 100 mg was planned. With this subsequent design, a two-sided α of 5% could be maintained for all three analyses. The estimates for progression were taken from the results of the previous study,^26^ where the participants in the DOX 200 mg group showed a progression in 4.9% of cases after 24 months whereas the placebo participants showed a progression in 55.6% of cases. Based on the assumption that the standardized and intensified hygiene measures in this trial would result in a stronger impact of this intervention in both treatment and placebo arms, a smaller difference between DOX and placebo was chosen to verify the added benefit of DOX. To account for this influence, the progression in the placebo group was assumed to be 25% instead of 55%. Based on these assumptions, there was a power of 95% for DOX 200 mg to show superiority to placebo and subsequently a power of 81% for DOX 100 mg versus placebo when 84 participants were included per treatment arm. With an estimated dropout rate of 30%, the final sample size was determined to be 120 participants per treatment arm.

### Treatment and assessment of adverse events.

Prior to enrollment and at the start of treatment, the trial procedures and potential side effects of DOX were explained to the trial participants. Directly observed treatment was implemented, with daily tablet intake supervised by a research team.^7^ Adverse events (AEs) were assessed and described during the first 4 months after treatment onset using the following terms: occurrence of AE, intensity of AE (grade 0, none; grade 1, mild; grade 2, moderate; grade 3, severe), serious AE (SAE), relation to treatment (definite, probable, possible, remote, not related), and outcome of AE (restored, improved, unchanged, deteriorated, death, unknown, overcome by sequelae, and intervention). Serious adverse events were assessed for the whole trial period of 24 months and after awareness by the research team, directly reported to the Tanzania Medicines and Medical Devices Authority and to the National Health Research Ethics Committee. MedDRA version 23.1 was used for coding of AEs and SAEs.

### Trial-specific measures.

Filarial LE patients were defined as people who had or had had nontraumatic progressive and evolving swelling of the lower limb(s) or leg(s), with nonfilarial causes ruled out as far as possible by a thorough medical examination.

#### LE staging.

Lymphedema-affected participants were selected based on the LE staging scale, which stages the legs from 1 to 7, as described by Dreyer et al.^31^ The trial team revisited study participants diagnosed with putative stage 1 LE early the next morning for confirmation that the swelling had disappeared overnight. Lymphedema staging was done during the screening, at baseline, and at 6-, 12-, 18-, and 24-month visits.

#### Clinical photography.

At baseline and at the 6-, 12-, 18-, and 24-month follow-up time points, digital clinical photographs of both legs of the trial participants were taken. The distance, lighting, and background were standardized for each participant, and every effort was made to ensure comparability. The photographs were stored as digital images.

#### Morbidity management and disability prevention.

Study participants were introduced to a program of cleaning the affected limbs based on the principles outlined in the booklet “New Hope for People with Lymphoedema.”^32^ Prior to screening and enrollment, all study participants were trained on how to use a standardized protocol of leg care, hygiene, and management that involved washing the limbs and the use of a diary for recording ADL attacks. The morbidity management program involved the following: cleaning of the affected leg(s) daily with soap and water, keeping the affected leg(s) dry, clipping the nails, applying topical antibacterial and antifungal creams to open sores, toe webs, nails, and sides of the feet every night, regular elevation of the affected leg when in a resting position, limb exercises as instructed, and encouraging and monitoring the use of appropriate footwear. Follow-ups and refresher training were offered to all trial participants at 4, 6, 12, 18, and 24 months after treatment. A questionnaire to assess leg care and hygiene was administered to each trial participant at baseline and at 4, 6, 12, 18, and 24 months after treatment.

#### Acute ADL assessment.

Questionnaires were carried out regarding previous experiences of ADL attacks, including swelling of the lymph nodes, feverishness, swelling of the legs, and the peeling off of the skin at the recession of the ADL attack. The questionnaire was administered at screening and baseline visits, during treatment, and every 2 months after treatment until 24 months to keep the recall bias as small as possible.

#### QoL assessments.

Quality of life assessments were performed at baseline and at 12 and 24 months using the 12-item WHO Disability Assessment Schedule (WHODAS 2.0) questionnaire.^33^ The WHODAS tools are in the English language, but they were administered in Kiswahili. Each of the 12 items can be answered with “none” (0), “mild” (1), “moderate” (2), “severe” (3), and “extreme” (4). The scores assigned to each item are summed at the end and converted into a metric ranging from 0 to 100, where 0 stands for “no disability” and 100 stands for “full disability.”

#### Circumference and volume measurement of legs.

A photo digital scanner tool known as the LymphaTech^®^^7^^,^^26^^,^^34^^,^^35^ and the classical tape measure tool were used to measure the limb volume and circumference of trial participants with LE. With the Lymphatech^7^ scanner tool, duplicate measurements were taken for each leg below the knee for volume and circumference. With the tape measure tool, duplicate leg circumference measurements were taken for each leg at 10 cm posterior to the tip of the large toe and at 12 cm, 20 cm, and 30 cm from the sole of the foot.^26^ Measurements using both the Lymphatech and tape measure methods were done at baseline and at 6, 12, 18, and 24 months after treatment onset.

#### Laboratory examinations.

Circulating filarial antigen (CFA) tests were done at the health facility with venous blood collected into ethylenediaminetetraacetic acid-containing monovettes using the Alere FTS (Alere Scarborough, Inc., Scarborough, ME). Briefly, 75 *μ*L of whole blood sample was added to the sample application pad of the FTS, and results were read after 10 minutes by two independent readers. Sedgewick and Giemsa/filter microfilarial counts were done for participants who were FTS positive. Counting was done using 100 *μ*L capillary blood and 3% acetic acid. The number of microfilariae (MF) counted was recordes as MF per milliliter (mL).^36^ These tests were repeated at 6, 12, and 24 months after the start of treatment.

To ensure participants’ safety at baseline, venipuncture was performed to assess platelet counts, neutrophil counts, and hemoglobin levels as well as transaminases, creatinine, and bilirubin. Transaminases were also checked before treatment number 22 and on the last day of treatment. Enrolled participants who had abnormal results before treatment number 22 had treatment stopped. Additionally, biochemistry tests were done for all participants at the end of the 6-week treatment.

Urine tests were done using Mission^®^ (ACON Laboratories, Inc., San Diego, CA) urinalysis reagent strips. Furthermore, pregnancy tests were done for all females below 55 years of age during screening, at baseline, prior to the first treatment, and after 14, 28, and 42 days of treatment as well as at the 2-, 6-, 12-, and 24-month follow-up time points. The human chorionic gonadotropin-accurate Excel Biotech^®^ (Thane, Maharashtra, India) pregnancy test kit was used to perform these tests at all time points.

### Follow-ups.

Follow-ups were undertaken every 2 months until 24 months after treatment onset. Major follow-ups within the trial protocol were scheduled at 6, 12, 18, and 24 months, and all the baseline procedures/measurements were repeated at these time points.

## STATISTICAL ANALYSES

REDCap^®^ (Research Electronic Data Capture) was used to build up the database for this trial.^37^^,^^38^ All data were entered on-site using double data entry, whereas the data management was located at the University Hospital Bonn, Germany, where the Institute for Medical Biometry, Informatics and Epidemiology hosts the REDCap server.

The following algorithm for comparisons of LE staging in participants with either one or both legs affected was used: 1) if only one leg was LE affected, this leg was analyzed; 2) if both legs were affected, one leg with stage 1–3 and the other leg with stage 4–7, the leg with the lower stage was chosen for analysis; 3) if both legs were affected with stage 1–3, the leg with the higher stage was chosen for analysis; and 4) if both legs were affected equally (same stage), one of them was chosen randomly for analysis.

**Figure 1.**
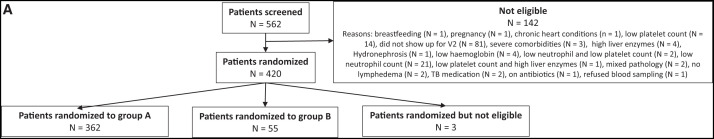

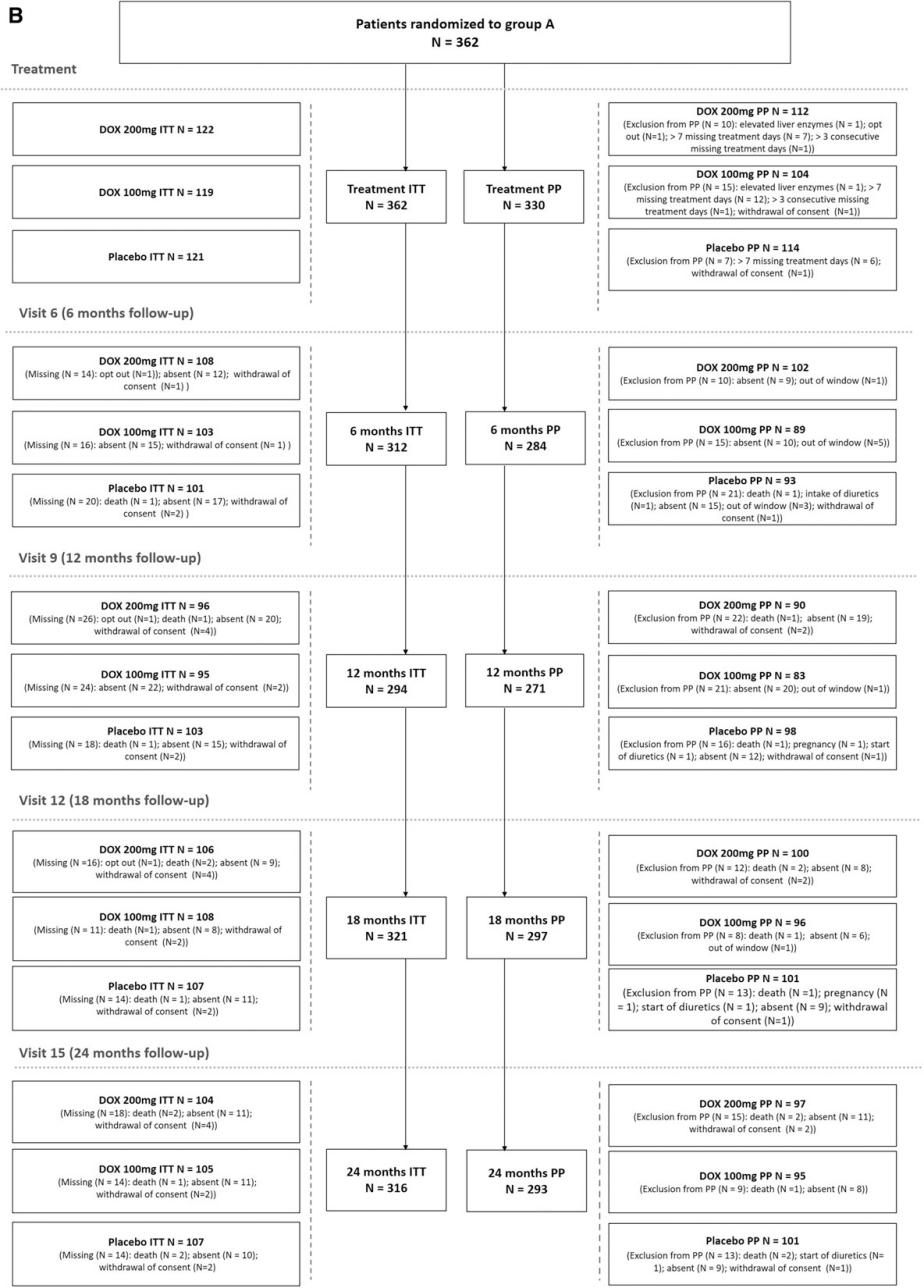
(**A**) Flowchart: recruitment and randomization. Of the 420 eligible participants, 362 were separately randomized for group A (lymphedema ≤ stages 1–3) and 55 for group B (LE stages 4–6). During data cleaning, it became apparent that three of the participants should not have been included because of diuretic use. Because this exclusion criterion was not chosen for safety purposes but because of the influence of the drugs on the edema, it was decided in consultation with the data safety and monitoring board before unblinding of the study to exclude the data of these three participants from all but the safety analyses. (**B**) Flowchart (see next page): treatment allocation. All group A participants were randomized into one of the three treatment arms and followed up over a period of 24 months. The graph also depicts the number of participants belonging to the intention-to-treat (ITT) and/or per-protocol (PP) analysis sets and the reason for absence or exclusion from PP analysis. DOX = doxycycline; TB = tuberculosis; V2 = visit 2.

In consultation with the data safety and monitoring board of this trial, three analysis sets were established before unblinding: 1) the safety set, which includes all participants randomized; 2) the intention-to-treat (ITT) set, which includes all participants correctly randomized and present for the respective follow-ups; and 3) the per-protocol (PP) set, which includes all the participants that completed the treatment per protocol, which were seen within the window of the respective follow-up visit, and which did not have any medical condition or treatment that would exclude them from the group at that time point (e.g., pregnant women). Four PP sets were established for the three major follow-up visits at 6, 12, 18, and 24 months. The safety set was used for the analysis of AEs and SAEs only, the ITT set was used for all analyses, and the PP sets were used for the univariate and bivariate analyses of the main outcome parameter to confirm the ITT analysis. The decisions on the allocations to the various analysis sets can be seen in the flow chart in Figure 1B.

The statistical analyses were performed using SAS version 9.4 (SAS Institute, Inc., Cary, NC). Descriptive statistics of continuous variables are presented as the mean ± standard error of the mean for normally distributed variables and median (interquartile range) for nonnormally distributed variables. Categorical variables are presented as numbers and percentages. For continuous variables, an analysis of variance or Kruskal-Wallis test was used to show differences between treatment groups at baseline. For all categorical variables, the Fisher exact test was used when possible to assess treatment differences. The statistical significance was defined as *P* <0.05.

Mixed-effects models with binary outcomes for progression, improvement, and hygiene status were used (PROC GENMOD). Effects are presented as odds ratios (OR) with 95% CI. We used a linear mixed-effects model with WHODAS as the outcome and presented the estimate (*β*) with 95% CI. Predictor variables for the mixed-effects models included sex, age, weight, treatment group, and region, as well as the time-dependent variables of ADL during the previous 6 months, LE stage, hygiene status, rainy season during the previous 6 months, and other leg affected.

Based on a reviewer’s comment, a subanalysis was done with consideration of both legs of a patient if they were affected at baseline. For this subanalysis, the same models were used for progression and improvement, but in cases where both legs were affected at baseline, a nested effect was used to account for the dependency of both legs in one participant.

For analysis of time to first occurrence of ADL, we plotted the Kaplan-Maier curve and used the log rank test to show a difference between treatments. To represent the occurrence of all ADLs, we used the approach of Anderson and Gil,^39^^,^^40^ which generates a Cox model formulated in terms of increments in the number of events along the time line, and the effects are presented as hazard ratios (HR) with 95% CI.

## RESULTS

Study participants (*N* = 562) were initially screened for participation in the RCT. A total of 420 study participants who met the eligibility criteria were then allocated into two groups (group A: LE stages 1–3; group B: LE stages 4–6) as illustrated in Figure 1A. In the following sections, only the results for group A are described and discussed. Results for the smaller group B will be published separately.

In group A (*N* = 362), study participants were randomly assigned to receive different doses of DOX (200 mg or 100 mg) or a placebo. The first participant was treated on September 25, 2018, and the 24-month follow-up of the last participant was carried out on September 24, 2021. The presence or absence of the participants during the respective follow-ups is shown in Figure 1B.

### Baseline data (Table 1).

The mean age of study participants was 51.3 ± 0.6 years, with a higher mean age among study participants allocated to the placebo group (*P* = 0.0197). Female study participants comprised at least two-thirds (67.1%; *n* = 243) of the total study participants with no difference among the treatment groups.

**Table 1:** Basic information of the study participants and area

Parameter	Unit	DOX 200 mg	DOX 100 mg	Placebo	Total	*P*-Value
Sex						
Female	*N* (%)	79 (64.8%)	80 (67.2%)	84 (69.4%)	243 (67.1%)	0.7347*
Male	*N* (%)	43 (35.2%)	39 (32.8%)	37 (30.6%)	119 (32.9%)	
Age (years)	*N*	122	119	121	362	0.0197^†^
	Mean ± SEM	51 ± 1	49.6 ± 1	53.3 ± 0.9	51.3 ± 0.6	
	95% CI of the mean	[48.9; 53]	[47.7; 51.5]	[51.6; 55.1]	[50.2; 52.4]	
	Min–max	15–65	18–65	20–65	15–65	
Region	Lindi	93 (76.2%)	89 (74.8%)	90 (74.4%)	272 (75.1%)	0.9526*
	Pwani	29 (23.8%)	30 (25.2%)	31 (26.0%)	90 (24.9%)	
Area	Rural	109 (89.3%)	105 (88.2%)	101 (83.5%)	315 (87%)	0.3665*
	Urban/semiurban	13 (10.7%)	14 (11.8%)	20 (16.5%)	47 (13%)	
Body weight (kg)	*N*	122	119	121	362	0.916^†^
	Mean ± SEM	60.2 ± 1.3	60.9 ± 1.3	60.8 ± 1.3	60.6 ± 0.7	
	95% CI of the mean	[57.6; 62.8]	[58.4; 63.4]	[58.2; 63.4]	[59.2; 62.1]	
	Min–max	40.5–111.5	40–110	40–104	40–111.5	
Weight category						
≤50 kg	*N* (%)	29 (23.8%)	25 (21%)	27 (22.3%)	81 (22.4%)	0.8892*
>50 kg	*N* (%)	93 (76.2%)	94 (79%)	94 (77.7%)	281 (77.6%)	
No. of years lived in area of endemicity	*N*	122	119	121	362	0.4155^†^
	Mean ± SEM	44.7 ± 1.3	43.8 ± 1.4	46.4 ± 1.5	45 ± 0.8	
	95% CI of the mean	[42.2; 47.3]	[41; 46.5]	[43.4; 49.4]	[43.4; 46.5]	
	Min–max	3–65	3–65	3–65	3–65	
Previous MDA rounds	*N*	98	95	94	287	0.2385^‡^
	Median, IQR	4; 3	3; 2	4; 2	4; 3	
	95% CI of the median	[3; 4]	[3; 4]	[3; 4]	[3; 4]	
	Min–max	1–15	1–15	1–15	1–15	
Filarial test strip						
Negative	*N* (%)	121 (99.2%)	118 (99.2%)	120 (99.2%)	359 (99.2%)	1.0*
Positive	*N* (%)	1 (0.8%)	1 (0.8%)	1 (0.8%)	3 (0.8%)	
Microfilariae						
Negative	*N* (%)	0 (0%)	1 (100%)	1 (100%)	2 (66.7%)	1.0*
Positive	*N* (%)	1 (100%)	0 (0%)	0 (0%)	1 (33.3%)	

DOX = doxycycline; IQR = interquartile range; MDA = mass drug administration; Min–max = minimum–maximum; SEM = standard error of the mean.

* Fishers’ exact test.

^†^ Analysis of variance.

^‡^ Kruskal-Wallis test.

The geographic distribution of study participants indicated that more study participants were enrolled from the Lindi region (75.1%; *n* = 272) than from the Pwani region. Further stratification of study participants by residency areas showed that the majority (87%; *n* = 315) were from rural areas. In both regions and areas of residency, no differences were discerned regarding the distribution of participants among the treatment groups at study onset. Similarly, no disparities were observed among the three treatment groups with regard to baseline measures such as body weight, years of residency in areas of endemicity, and previous rounds of MDA. Remarkably, there were only three participants positive for FTS, and only one of those was MF positive.

Table 2 shows the baseline data for the trial-specific measures of LE staging, episodes of ADL, adherence to hygiene measures, and QoL. There was no difference between the randomized groups in relation to the numbers of years having LE, the number of affected legs, or the stage of both legs.

**Table 2:** Baseline data for trial-specific measures

Parameter	Unit	DOX 200 mg	DOX 100 mg	Placebo	Total	*P*-Value
No. of years having LE	*N*	102	94	96	292	0.6424*
	Median; IQR	26; 20	21.5; 23	25; 23	24.5; 23	
	95% CI of the median	[24; 29]	[19; 26]	[20; 30]	[21; 26]	
	Min–max	1–49	1–49	2–49	1–49	
No. of legs affected						
Only one leg	*N* (%)	83 (68%)	96 (80.7%)	92 (76%)	271 (74.9%)	0.0763^†^
Both legs	*N* (%)	39 (32%)	23 (19.3%)	29 (24%)	91 (25.1%)	
Both legs have the same stage						
No	*N* (%)	10 (25.6%)	12 (52.2%)	16 (55.2%)	38 (41.8%)	0.0254^†^
Yes	*N* (%)	29 (74.4%)	11 (47.8%)	13 (44.8%)	53 (58.2%)	
LE staging of study leg						
1–Swelling is reversible	*N* (%)	0 (0%)	2 (1.7%)	3 (2.5%)	5 (1.4%)	0.0323^†^
2–Swelling is not reversible	*N* (%)	58 (47.5%)	67 (56.3%)	48 (39.7%)	173 (47.8%)	
3–Presence of shallow skin folds	*N* (%)	64 (52.5%)	50 (42%)	70 (57.9%)	184 (50.8%)	
LE staging of other leg						
1–Swelling is reversible	*N* (%)	1 (0.8%)	2 (1.7)	1 (0.8)	4 (1.1)	0.1635^‡^
2–Swelling is not reversible	*N* (%)	16 (13.1%)	8 (6.7)	7 (5.8)	31 (8.6)	
3–Presence of shallow skin folds	*N* (%)	18 (14.8%)	8 (6.7)	10 (8.3)	36 (9.9)	
4–Presence of skin knobs	*N* (%)	0 (0%)	1 (0.8)	1 (0.8)	2 (0.6)	
5–Presence of deep skin folds	*N* (%)	0 (0%)	1 (0.8)	1 (0.8)	2 (0.6)	
6–Presence of mossy lesions	*N* (%)	4 (3.3%)	3 (2.5)	9 (7.4)	16 (4.4)	
ADL attacks since:						
Missing answers	*N* (%)	29	40	36	105	0.5977^‡^
<1 year	*N* (%)	2 (2.2%)	2 (2.5%)	1 (1.2%)	5 (1.9%)	
≥1–5 years	*N* (%)	10 (10.8%)	9 (11.4%)	8 (9.4%)	27 (10.5%)	
≥5–10 years	*N* (%)	6 (6.5%)	9 (11.4%)	15 (17.6%)	30 (11.7%)	
≥10–15 years	*N* (%)	11 (11.8%)	5(6.3%)	8 (9.4%)	24 (9.3%)	
≥15–20 years	*N* (%)	8 (8.6%)	9 (11.4%)	5 (5.9%)	22 (8.6%)	
≥20 years	*N* (%)	56 (60.2%)	45 (57%)	48 (56.5%)	149 (58%)	
Last ADL attack (months before screening)	*N*	97	95	95	287	0.0494*
	Median, IQR	7; 11	13; 12	13; 12	13; 13	
	95% CI of the median	[7; 13]	[7; 14]	[13; 15]	[7; 13]	
	Min–max	1–52	1–160	1–170	1–170	
Duration of last ADL attack (days)	*N*	122	116	120	358	0.1832*
	Median, IQR	4; 2	3; 2	4; 2	4; 2	
	95% CI of the median	[4; 4]	[3; 4]	[3; 4]	[3; 4]	
	Min–max	1–30	1–14	2–30	1–30	
No. of attacks within the last year (only patients who had attacks)	*N*	79	72	67	218	0.7273*
	Median, IQR	2; 2	2; 2	2; 1	2; 2	
	95% CI of the median	[2; 2]	[1; 2]	[1; 2]	[2; 2]	
	Min–max	1–4	1–5	1–6	1–6	
Overall hygiene: limb kept washed and clean						
Missing	*N* (%)	10	12	15	37	0.3541^†^
No	*N* (%)	4 (3.6%)	6 (5.6%)	2 (1.9%)	12 (3.7%)	
Yes	*N* (%)	108 (96.4%)	101 (94.4%)	104 (98.1%)	313 (96.3%)	
WHODAS 2.0 score	*N*	119	116	121	356	0.3169^§^
	Mean ± SEM	3.61 ± 0.47	3.01 ± 0.74	4.73 ± 1	3.79 ± 0.47	
	95% CI mean	[2.31; 4.9]	[1.55; 4.48]	[2.76; 6.71]	[2.87; 4.72]	
	Min–max	0–45.9	0–52.1	0–81.3	0–81.3	

ADL = adenolymphangitis; DOX = doxycycline; IQR = interquartile range; LE = lymphedema; Min–max = minimum–maximum; SEM = standard error of the mean.

^*^ Kruskal-Wallis test.

^†^ Fishers’ exact test.

^‡^ Chi-square test.

^§^ Analysis of variance.

Regarding the study leg (the leg that was chosen for all primary analyses), about half (50.8%; *n* = 184) of the study participants had the presence of shallow skin folds (stage 3), 47.8% (*n* = 173) had a swelling that was not reversible (stage 2), and only 1.5% (*n* = 5) had reversible swelling (stage 1). Despite the randomization of the trial participants, which was not done separately for the different stages, a slight imbalance was seen in the distribution of study leg LE stages (*P* = 0.0323), with more participants with stage 3 than with stage 1 or 2 in the placebo group (57.9%; *n* = 70) compared with the DOX 200 (52.5%; *n* = 64, *P* = 0.4398) and DOX 100 (42%; *n* = 50, *P* = 0.0199) groups. Additionally, a significant difference was found between the groups when looking at the participants in whom both legs were affected at baseline (*P* = 0.0254) with more participants in the DOX 200 group having the same stage.

More than three-quarters (79.0%; *n* = 257) of the study participants were able to recall their ADL history. There was a slight difference seen in the median number of months of the most recent ADL attacks between study participants assigned to different treatment groups (*P* = 0.0494). Study participants who were assigned to the DOX 200 group had the lowest median number of months since the most recent ADL attack, with 7 months, whereas those in the DOX 100 and placebo groups had a median of 13 months since the last ADL episode. There were no differences between the treatment groups in terms of occurrence of the first ADL attack, duration of the last attack, and the frequency of attacks in the previous year.

At baseline, after the participants received their first hygiene training during the screening visit, the majority of participants (96.3%; *n* = 313) ensured that their limbs were kept washed and clean. with no variation observed in terms of the randomized treatment groups (*P* = 0.3541).

For the WHODAS 2.0, the scores for each of the 12 items were summed and converted into a metric ranging from 1 to 100, where a low value indicates a good QoL (0 = no disability) and a high value indicates a bad QoL (100 = full disability).^33^ In this trial, most of the participants reported a very good QoL at baseline, with a mean of 3.79 ± 0.47 for all three groups, probably reflecting good coverage by the national MMDP program already before study onset.

### Lymphedema progression and improvement.

**Figure 2.**
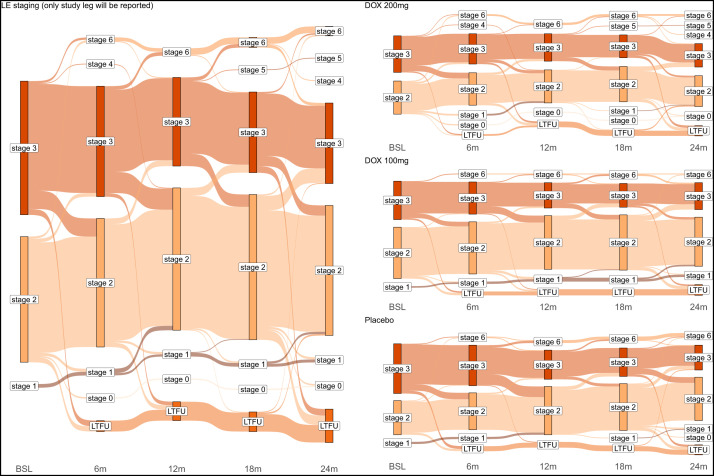
Sankey diagram of stage changes over the whole trial period. On the left side, stage changes are shown for all groups together; on the right side, they are shown separately for each treatment arm. The diagram represents the data for the intention-to-treat collective. BSL = baseline; DOX = doxycycline; LE = lymphedema; LTFU = lost to follow-up.

**Figure 3.**
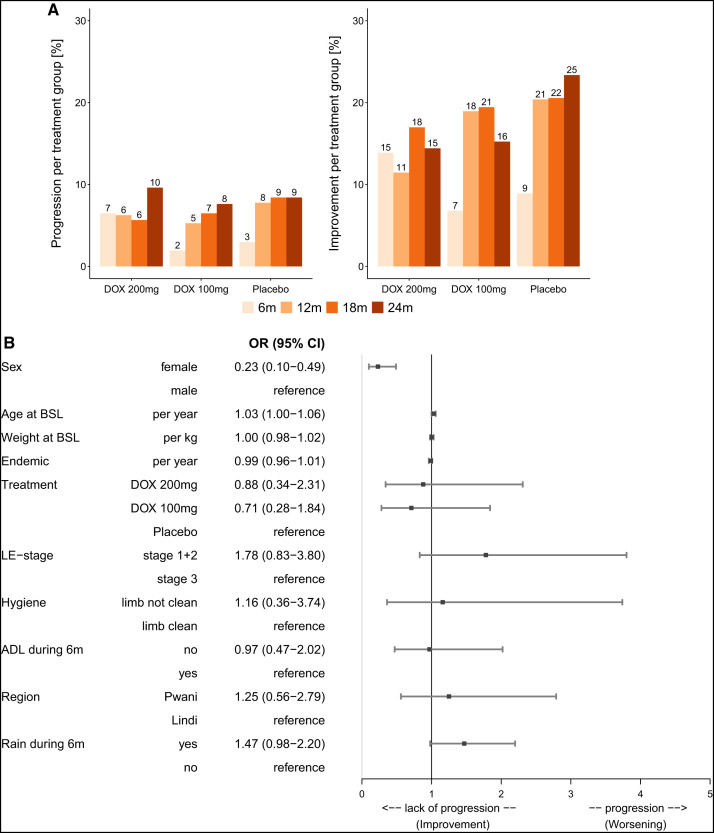

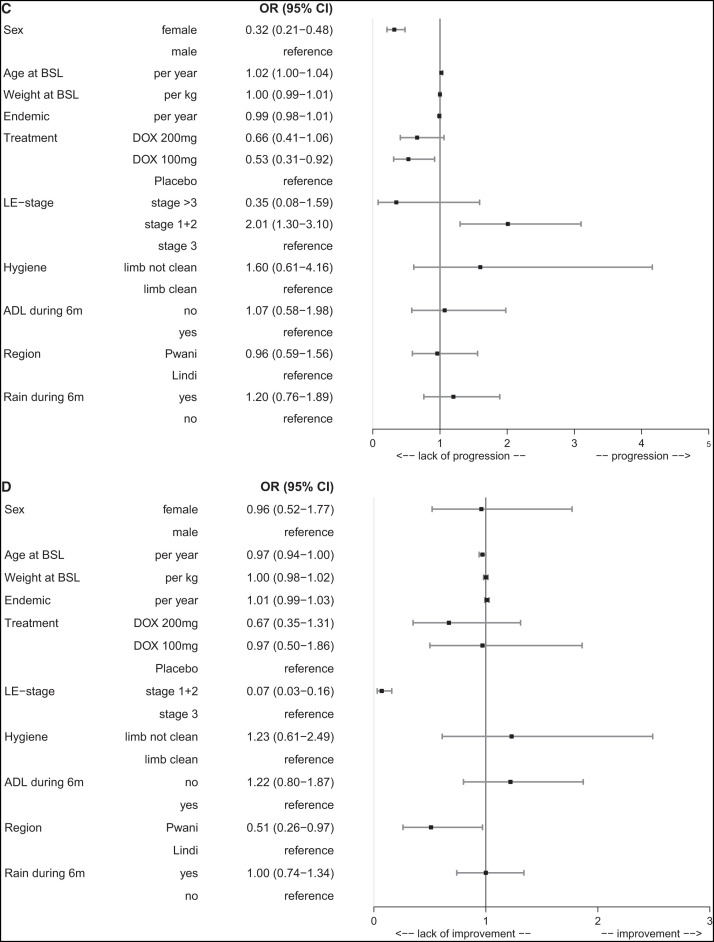
(**A**) Stage progression and stage improvement. In the left diagram, the percentage as well as the number of participants who had progression (worsening) of their lymphedema (LE) is shown per follow-up and for each treatment separately; in the right diagram, the same is shown for improvement of LE. The diagrams represent the data of the intention-to-treat (ITT) collective. The percentages are always calculated for the total number of participants who were present at the particular follow-up and can therefore differ among the time points. (**B**) Forest plot: multivariable analysis for stage progression over time (including study leg only). The forest plot depicts the different covariables that were used in a multivariable logistic regression model (PROC GENMOD, SAS) for the outcome variable “progression.” The following baseline covariables were used for this model: sex (male/female), age, weight, years in area of endemicity, LE staging (stage 1 or 2/stage 3), treatment (DOX 200 mg/DOX 100 mg/placebo), and region (Pwani/Lindi). In addition, the following time-dependent covariables were used (with changes during the follow-up period taken into account): hygiene status (limb not clean/limb clean), acute adenolymphangitis (ADL) attack during the previous 6 months (no/yes), and more days of rainy season during the previous 6 months (yes/no). Effects are presented as odds ratios (OR) with 95% CI. (**C**) Forest plot (see next page): multivariable analysis for stage progression over time (including all affected legs at baseline). The forest plot depicts the different covariables that were used in a multivariable logistic regression model (PROC GENMOD, SAS) for the outcome variable “progression.” The following baseline covariables were used for this model: sex (male/female), age, weight, years in area of endemicity, LE staging (stage 1 or 2/stage 3), treatment (DOX 200 mg/DOX 100 mg/placebo), and region (Pwani/Lindi). In addition, the following time-dependent covariables were used (with changes during the follow-up period taken into account): hygiene status (limb not clean/limb clean), ADL attack during the previous 6 months (no/yes), and more days of rainy season during the previous 6 months (yes/no). Effects are presented as OR with 95% CI. (**D**) Forest plot: multivariable analysis for stage improvement over time (including study leg only). The forest plot depicts the different covariables that were used in a multivariable logistic regression model (PROC GENMOD, SAS) for the outcome variable “improvement.” The following baseline covariables were used for this model: sex (male/female), age, weight, years in area of endemicity, LE staging (stage 1 or 2/stage 3), treatment (DOX 200 mg/DOX 100 mg/placebo), and region (Pwani/Lindi). In addition, the following time-dependent covariables were used (with changes during the follow-up period taken into account): hygiene status (limb not clean/limb clean), ADL attack during the previous 6 months (no/yes), and more days of rainy season during the previous 6 months (yes/no). Effects are presented as OR with 95% CI. BSL = baseline; DOX = doxycycline; m = months.

Of the 316 participants present for the 24-month follow-up, overall only 27 (8.5%) showed progression (worsening), with 10/104 (9.6%) in the DOX 200 group and 8/105 (7.6%) in the DOX 100 group showing no differences in comparison with the 9/107 (8.4%) in the placebo group (*P* = 0.8133 and *P* = 1.0, respectively) (Figures 2 and 3A). On the other hand, 56/316 (17.7%) participants had an improvement at that time point, with 15/104 (14.4%) in the DOX 200 group, 16/105 (15.2%) in the DOX 100 group, and 25/107 (23.4%) in the placebo group (Figures 2 and 3B). Comparison of the DOX 200 or DOX 100 group to the placebo group did not reveal a statistical difference for this 24-month time point (*P* = 0.115 and *P* = 0.165, respectively). For the PP analysis of LE progression and improvement, see Supplemental Figure 3.

The greatest improvement in LE occurred in year 1, whereas progression showed an increase with time, especially in those randomized to the DOX 100 and placebo groups (Figures 2 and 3A).

There was a slight imbalance at baseline, with a higher number of LE stage 3 study participants in the placebo group (*P* = 0.0323; Table 2) (*P* = 0.4398 for DOX 200 versus placebo, *P* = 0.0199 for DOX 100 versus placebo, and *P* = 0.1219 for DOX 200 versus DOX 100). Since stage 3 LE inherently has a greater chance to improve than stage 2 LE (as one would have to document for stage 1 that the legs have no swelling in the morning before the participants got up, which was not possible in this trial), this resulted in slightly more improvement (Figures 2 and 3A) in the placebo group, though the difference in improvement between the placebo group and the DOX 200 or DOX 100 group after 24 months was not statistically significant in the univariate analyses (*P* = 0.115 and *P* = 0.1646, respectively).

Multivariate analysis of LE stage progression over time (Figure 3B) indicated that women tended to have less progression than men (OR: 0.23, *P* = 0.0002).

In the multivariate subanalysis (Figure 3C), which included all affected legs at baseline (Supplemental Figure 4A), the primary finding of women having less progression was confirmed. In addition, the primary trend that participants with stage 1 and 2 LE were more likely to have disease progression than those with stage 3 was significant in this analysis (OR: 2.01, *P* = 0.0016). Even more interesting is that DOX 100 participants showed significantly less progression than placebo participants (OR: 0.53, *P* = 0.0239), whereas a trend towards the same effect was observed for DOX 200 participants (OR 0.66, *P* = 0.0877). The effect remained significant when both DOX groups were combined in the model (OR: 0.6, *P* = 0.0201). The fact that there were significantly more participants with LE stage 1 and 2 in the DOX 100 group than in the placebo group at baseline could have led to the assumption that there might be more disease progression in the DOX 100 group, but since the exact opposite is the case, this underscores a treatment effect.

The multivariate analysis for improvement (Figure 3D) revealed that participants with LE stage 1 or 2 were 93% less likely to improve compared with participants with LE stage 3 (OR: 0.07, *P* <0.0001). Additionally, it was found that the participants from the Pwani region were 49% less likely to improve than the participants from the Lindi region (OR: 0.51, *P* = 0.0402).

The multivariate subanalysis for improvement (Supplemental Figure 4B) confirmed the results from the primary analysis regarding the dependency on the region and the fact that stage 3 legs were more likely to improve than legs with stage 1 or 2. It was also found that legs with a stage higher than 3 were less likely to improve than those with stage 3 (OR: 0.51, *P* = 0.0338). In addition, there was a significant difference between the DOX 200 and placebo groups, with less improvement in the DOX 200 group (OR: 0.66, *P* = 0.0184), most likely influenced by the effect of more legs with stage 1 and 2 in the DOX 200 group.

### Hygiene assessment.

**Figure 4.**
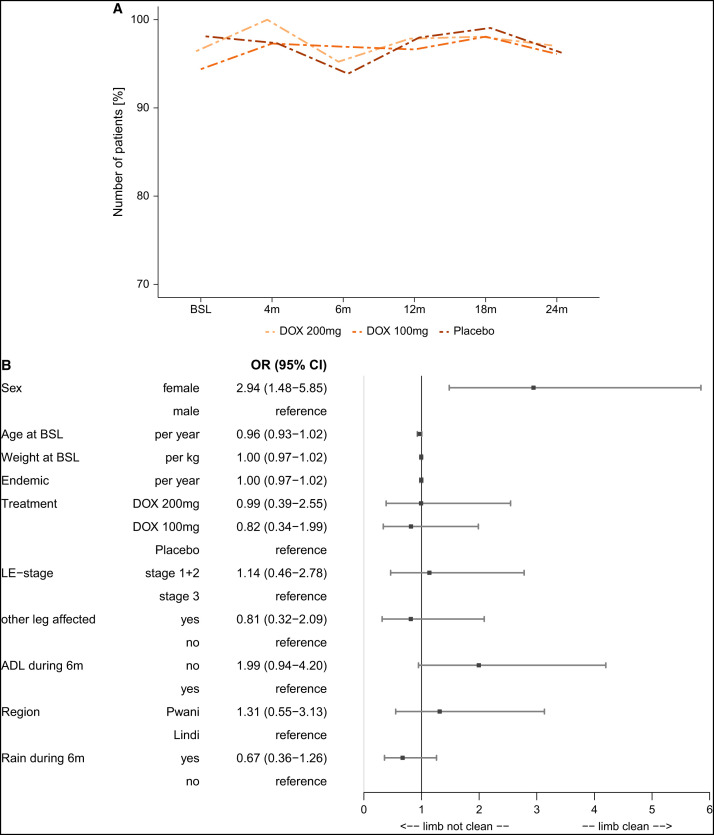
(**A**) Hygiene status: limb washed and clean. The graph shows the number of participants who had their limbs washed and clean at the six different time points of hygiene assessment. Dotted lines represent that not all participants were present for every assessment. (**B**) Forest plot: multivariable analysis for hygiene status over time. The forest plot depicts the different covariables that were used in a multivariable logistic regression model (PROC GENMOD, SAS) for the time-dependent outcome variable “hygiene status.” The following baseline covariables were used for this model: sex (male/female), age, weight, years in area of endemicity, lymphedema (LE) staging (stage 1 or 2/stage 3), treatment (DOX 200 mg/DOX 100 mg/placebo), and region (Pwani/Lindi). In addition, the following time-dependent covariables were used if the other leg was also affected (with changes during the follow-up period taken into account): acute adenolymphangitis (ADL) attack during the previous 6 months (no/yes) and more days of rainy season during the previous 6 months (yes/no). Effects are presented as odds ratios (OR) with 95% CI. BSL = baseline; DOX= doxycycline; m = months.

The hygiene assessment started already at a very high level, with 96.3% of the participants already having washed and cleaned their lower limbs, with no differences among the three treatment arms (Table 2 and Figure 4A). In the DOX 200 and placebo groups, there was a decline in hygiene status at 6 months, followed by subsequent improvement until 18 months. A minor decline was observed in all groups between 18 and 24 months (Figure 4A). However, it is worth noting that only a small subset of participants across all groups did not have washed and clean legs already at baseline.

The multivariate analysis (Figure 4B) indicated that women were more likely to have better limb hygiene than men (OR: 2.94, *P* = 0.0021). Interestingly, participants who, by clinical history, experienced an ADL attack during the 6 months prior to the hygiene assessment showed a trend to a higher probability for a not clean and washed limb (OR: 1.99, *P* = 0.069).

### Acute ADL attacks.

**Figure 5.**
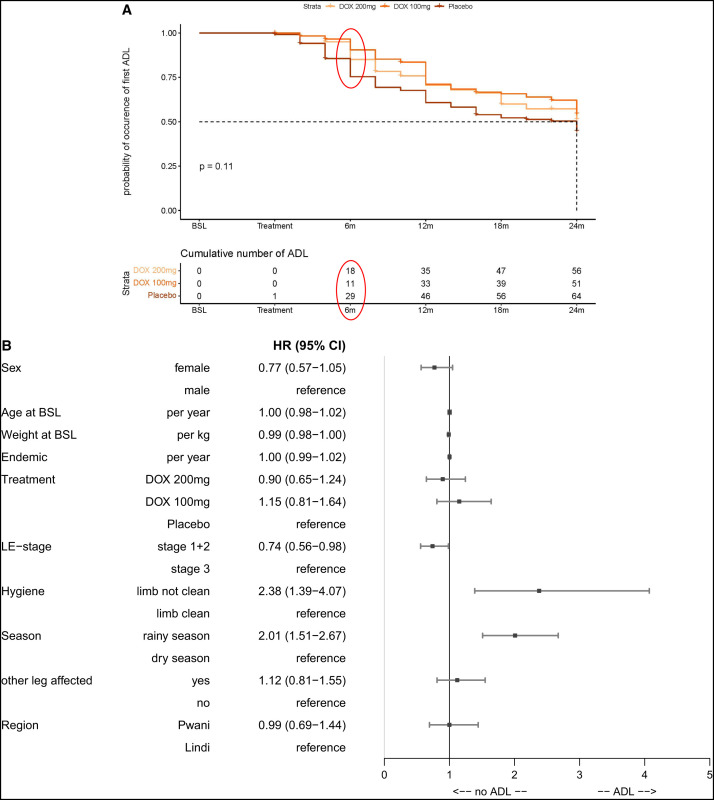
Acute adenolymphangitis (ADL). (**A**) Time to first attack after treatment start. The Kaplan-Meier curve shows the time to occurrence of the first attack after treatment onset. The graph is divided by treatment groups. At 6 months after treatment onset, there was a significantly higher number of participants in the placebo group who had already experienced an ADL attack than in the two doxycycline groups (red circles). The median survival time, i.e., time until ≥50% experienced an acute attack, was reached in the placebo group only at 24 months after treatment onset (dotted line). (**B**) Count model—multivariable analysis of ADL attacks over time. To take not only the first but the occurrence of all ADL attacks during the follow-up period of 24 months into account, the approach of Anderson and Gill,^35^^,^^36^ which generates a Cox model formulated in terms of increments in the number of events along the time line, was used. The forest plot depicts the different covariables that were used in a Cox model for the time-dependent count variable “ADL attack.” The following baseline covariables were used for this model: sex (male/female), age, weight, years in area of endemicity, lymphedema (LE) staging (stage 1 or 2/stage 3), treatment (DOX 200 mg/DOX 100 mg/placebo), and region (Pwani/Lindi). In addition, the following time-dependent covariables were used if the other leg was also affected (taking changes during the follow-up period into account): hygiene status (limb not clean/limb clean) and the season (rainy season/dry season) at the time of assessment. The effects are presented as hazard ratios (HR) with 95% CI. BSL = baseline; DOX = doxycycline; m = months.

A Kaplan-Meier curve (Figure 5A) shows the time to the first ADL attack after start of treatment. There was no difference between the three treatment groups over the whole study period of 24 months (*P* = 0.11; log rank test). However, the median “survival time” (time until 50% of the participants experienced the first attack) was reached in the placebo group only at 24 months. Even more interesting, over the first 6 months after treatment, the DOX groups experienced significantly fewer ADL episodes than the placebo group (*P* = 0.0043 overall, *P* = 0.0323 for DOX 200 versus placebo, *P* = 0.0012 for DOX 100 versus placebo; log rank test).

We analyzed the impact of several factors in relation to the occurrence and number of ADL attacks in a multivariate time-dependent Cox model, including sex, age, weight at baseline, years in the area of endemicity, treatment, LE staging, hygiene, seasonality, other leg affected, and region as covariables (Figure 5B). Strikingly, having the limb not clean increased the likelihood of an ADL attack by 2.38 (*P* = 0.0016) compared with having the limb clean. Interestingly, during the rainy season, participants were 2.01 (*P* <0.0001) times more likely to experience an ADL attack than in the dry season. Furthermore, participants with stage 1 or 2 had a lower chance for an ADL attack than participants with stage 3 (HR: 0.74; *P* = 0.038).

### QoL (WHODAS 2.0).

For the WHODAS 2.0, the scores for each of the 12 items were summed and converted into a metric ranging from 1 to 100, where a low value indicates a good QoL (0 = no disability) and a high value indicates a bad QoL (100 = full disability).^33^ The participants in this trial had started already with a very good QoL, as indicated by a lower score. This low score was maintained over the whole study period without differences among the treatment groups.

Upon dissecting the effect of the aforementioned factors on the QoL in a multivariate linear time-dependent analysis, we found that having no ADL attack in the 6 months prior to the QoL questionnaire led to a better QoL (estimate: –17.16; *P* <0.0001) than did having an ADL attack previously. Quite remarkably, in cases where both legs were affected by the disease, the participants tended to have a higher QoL score (indicating a worse QoL) over time than the participants with only one leg affected (estimate: 2.61; *P* = 0.004). Additionally, each additional kilogram of weight led to a higher QoL score (estimate: 0.06; *P* = 0.0232).

### Safety.

A total of 162 AEs were reported by 105 participants during treatment (Table 3) as well as in the period between treatment end and the 4-month follow-up (Supplemental Table 2), all of them graded with mild (grade 1, *n* = 144) or moderate (grade 2, *n* = 17) severity except for one SAE, which occurred during the 4-month period. Serious adverse events were documented and reported during the whole study period of 24 months. In total, five SAEs (deaths) occurred, all of them determined not to be related to treatment, as follows: 1) stroke in a 64-year-old man 4 months after treatment onset in the placebo group, 2) sepsis in a 32-year-old woman 10 months after treatment onset (8.5 months after treatment stopped) in the DOX 200 group, 3) unknown cause of death of a 52-year-old woman under chemotherapy for breast cancer at 18 months after treatment onset (16.5 months after treatment stopped) in the DOX 100 group, 4) unknown cause of death of a 55-year-old man at 18 months after treatment onset (16.5 months after treatment stopped) in the DOX 200 group, 5) stroke in a 57-year-old woman at 24 months after treatment onset (22.5 months after treatment stopped) in the placebo group.

**Table 3:** Most reported adverse events during treatment

Adverse Event	No. (%) of Events Reported for Group			Total
	DOX 200 mg	DOX 100 mg	Placebo	
Pyrexia	5 (38.5)	2 (15.4)	6 (46.2)	13
Headache	4 (36.4)	4 (36.4)	3 (27.3)	11
Increase in liver enzymes*	4 (36.4)	7 (63.6)	0 (0)	11
Vomiting	4 (44.4)	4 (44.4)	1 (11.1)	9
Abdominal discomfort/pain	3 (42.9)	3 (42.9)	1 (14.3)	7
Arthralgia	1 (25)	0 (0)	3 (75)	4
Diarrhea	2 (50)	1 (25)	1 (25)	4
Dizziness	1 (25)	3 (75)	0 (0)	4
Cough	2 (66.7)	0 (0)	1 (33.3)	3
Pain in extremity	0 (0)	1 (33.3)	2 (66.7)	3
Malaria	1 (33.3)	0 (0)	2 (66.7)	3
Nausea	1 (33.3)	1 (33.3)	1 (33.3)	3
Wound	0 (0)	2 (66.7)	1 (33.3)	3
Other^†^	11 (45.8)	4 (16.7)	9 (37.5)	24
Total	39 (38.2)	32 (31.4)	31 (30.4)	102

DOX = doxycycline.

* In one participant, all three enzymes (alanine transaminase [ALT], aspartate aminotransferase [AST], and gamma-glutamyl transferase [γGT]), in one participant, two enzymes (AST, ALT), and in the other participants, only one of the three enzymes measured were increased above the limit of 2 times the upper normal (AST [*n* = 1], ALT [*n* = 2], γGT [*n* = 6]), 35 participants did not have blood sampled at day 22 during treatment, 18 participants stopped treatment before or on day 22, and 17 participants refused to have blood sampled even after they were informed again about the safety reasons and decided to continue treatment without blood sampling. Fifty-eight participants did not have blood sampled at the end of treatment, 21 stopped treatment before the end of treatment, and 37 participants refused or were absent for blood sampling even after they were informed again about the safety reasons.

^†^ Other – occurrence <3 (i.e., asthenia, influenza, malaise, pruritus, burning sensation, chest pain, constipation, caries, dysuria, groin pain, hypoesthesia, leprosy, decrease in libido, lymphadenitis, lymphangitis, oral pain, peripheral swelling, rash, somnolence, urinary tract infection).

## DISCUSSION

### Overview of key results.

Our study aimed to determine whether a 6-week course of doxycycline 200 mg daily added to the WHO standard hygiene-based management regimen for filarial LE could effectively halt or reverse the progression of LE among people living in the areas of endemicity of the Lindi and Pwani regions in Tanzania. The study consisted of 362 participants between the ages of 15 and 65 years with a mean age of 51.3 years (median: 53 years) and a majority of women (67.1%). The principal findings were as follows. 1) By the end of the 2-year study, LE of the limb had progressed only in 8.5% of the patients compared with baseline. This underscores the effectiveness of the measures taken and the value of stringent follow-up. Lymphedema stages actually improved in 17.7% of participants and did not change in 73.8%. 2) In the primary analysis that included only one affected leg per participant, there were no significant differences among the treatment regimens (DOX 200, DOX 100, and placebo) in the outcomes of either “halted progression” or “stage improvement”; all effects were seen equally in the three treatment groups. 3) In a subanalysis which included all legs affected at baseline, LE was more likely to progress in the legs of participants from the placebo group than in the legs of participants from the DOX groups (significant for DOX 100 and a trend for DOX 200). 4) Although the study did not find significant differences in hygiene (assessed cleanliness) among the three treatment groups, it did observe that women had better limb hygiene than men. 5) During the first 6 months after treatment onset, the DOX groups experienced significantly fewer ADL episodes than the placebo group. The median time until 50% of participants experienced the first attack was reached only in the placebo group after 24 months. 6) Participants tended to have higher QoL scores (indicating a worse overall QoL) when they experienced an acute ADL episode in the 6 months prior to the questionnaire, when both legs were affected by the disease at baseline, or with each additional kilogram of body weight.

### Doxycycline therapy outcome.

Several previous studies have shown that DOX is highly effective in the clearance of LF infections,^21^^,^^25^^,^^41^^–^^44^ and a few have also demonstrated the effectiveness of the drug in reducing or halting the progression of LE.^26^^,^^45^^–^^47^ The present study that monitored patients closely for 2 years after a 6-week course of DOX 200 mg, DOX 100 mg, or placebo failed to show a difference in the primary analysis that included only one affected leg per participant between these treatment groups in halting the progression of LE in patients. However, in a subanalysis that included all affected legs at baseline, participants from the DOX 100 group were significantly more likely to have a halt of progression than the placebo group. The DOX 200 group showed a trend in the same direction. This subanalysis was originally not planned as primary analysis and was done based on a reviewers’ comment. The outcome of the subanalysis will need further investigation, especially when the results from Tanzania are combined with those from four other studies.^27^^–^^29^^,^^48^

Two explanations, both very important, likely contribute to these findings. The first is suggested by the remarkably low rate of LE progression seen not only in patients receiving just placebo (8.5%) but also in patients on DOX (either dose: 7% and 9%), different from earlier studies. This finding suggests that strict adherence to the hygiene protocol maintained in this study (which was more stringent than that in prior investigations of DOX versus placebo) alone might have been able to halt progression of the filarial lymphedema. Although the earlier placebo-controlled RCTs had included hygiene training at the beginning of the trials that was similar to standard MMDP practices, it was not as stringent as the rigorous training and retraining offered at baseline and at 4, 6, 12, 18, and 24 months in the present study. A second important and likely explanation for not finding all the expected differences in the DOX and placebo outcomes is that the previous studies took place 10–15 years earlier in areas that were then experiencing considerably more ongoing transmission of filarial infection and active incidence of disease; it may well be that the 6-week DOX treatment in those studies resulted in the killing of incoming larvae and developing adult worms that likely triggered lymphatic inflammation and worsened LE in these patients. By contrast, in the present study, very few patients were CFA positive (Supplemental Table 3), suggesting an epidemiologic situation much closer to interrupted transmission, where patients are no longer exposed continually to new infections. The absence of such stimuli might explain why, in the present study, DOX appeared to result in less benefit than in earlier studies.

### Hygiene measures.

Our research findings also align with other studies that indicated the positive effects of hygiene, education, and self-care on limiting morbidity, reducing disability, and enhancing the QoL in affected individuals.^49^^–^^53^ Having an unclean limb increased the risk of an ADL attack by a factor of 2.36 (*P* = 0.0016), consistent with the findings from a review on the impact of hygiene-based interventions.^54^ Moreover, it revealed that participants who reported having had an ADL attack within the 6 months prior to the hygiene assessment had a higher likelihood of having an unclean and unwashed limb (OR: 2.2, *P* = 0.0404), again in line with studies showing that participating in hygiene-based LE management was linked to a reduced incidence of ADL and a lower percentage of patients reporting at least one episode of ADL during follow-up.^54^ The importance of implementing an essential minimum package of self-care measures has also been highlighted in other studies demonstrating that basic LE management was associated with a reduction in ADL incidence.^50^^,^^55^^,^^56^ Clearly, there is a need for patients with LE to be informed about the MMDP services in their countries, even in situations where it is known that not all of those infected will consistently practice good hygiene measures.^57^

### Acute ADL attacks.

Because of the potential for increased pain, discomfort, exacerbation of swelling, injury risk, compromised functionality, skin complications, and negative psychological impact, ADL attacks are a major concern for individuals with LF and LE.

In our study, we investigated the occurrence and management of ADL episodes among participants who received different treatments. During the initial 6 months after receiving treatment, there were significantly fewer participants in the two DOX treatment arms who reported a new ADL episode than in the placebo group (*P* = 0.0043). Other studies have also shown that supervised home care and regular clinic attendance are necessary to ensure clinical benefits such as reduced limb volume and fewer ADL attacks.^58^^–^^60^

Earlier observations from a study carried out in the Rufiji district of southeastern Tanzania revealed a noteworthy pattern in the seasonal incidence of ADL, with an increase during the long rains that typically take place from March to May^61^ and again in August, after a second rainy season. These observations suggest a potential link between rainfall and ADL incidence,^61^ which could be confirmed by our study, where in the multivariate analysis, participants were 2.01 times more likely to experience an ADL attack during the rainy season than in the dry season (*P* <0.0001). These findings underscore the complexity of the triggers of ADL episodes and the need for further research to better understand and mitigate the factors contributing to their occurrence.

### Quality of life.

**Figure 6.**
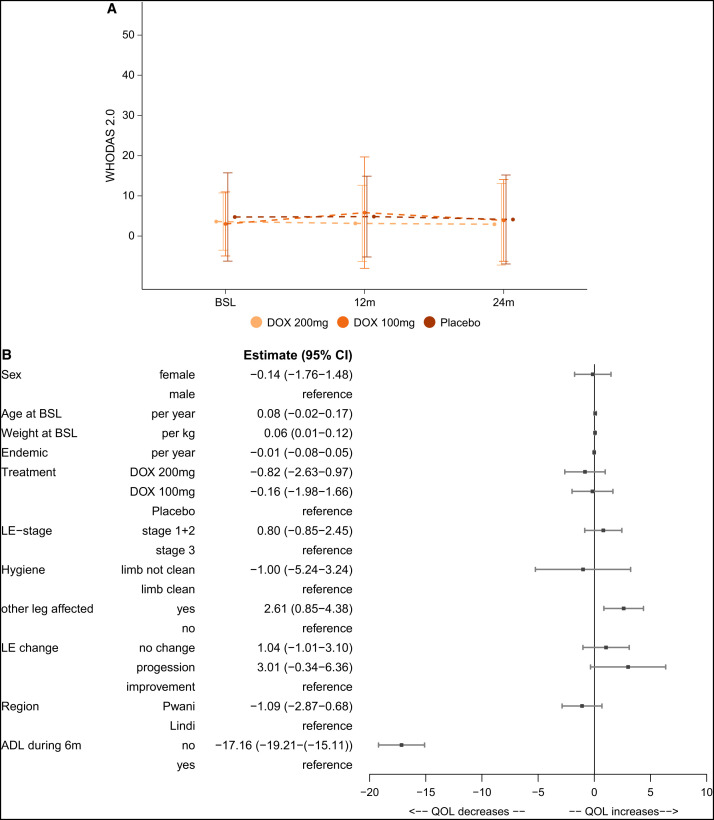
Quality of life. (**A**) WHODAS 2.0. The graph shows the mean and standard error of the mean of the WHODAS 2.0 score at the three different time points split between the treatment groups. Dashed lines between the scores indicate that not all participants completed the WHODAS 2.0 at each time point, but the mean represents all participants who were present at the respective follow-up visits. (**B**) Multivariable analysis for WHODAS 2.0 changes over time. A linear mixed-effects model was used with the WHODAS 2.0 score as the outcome. The forest plot depicts the different covariables that were used in the linear mixed-effects model. The following baseline covariables were used for this model: sex (male/female), age, weight, years in area of endemicity, lymphedema (LE) staging (stage 1 or 2/stage 3), treatment (DOX 200/DOX 100/placebo), and region (Pwani/Lindi). In addition, the following time-dependent co-variables were used if the other leg was also affected (with changes during the follow-up period taken into account): LE change (no change/progression/improvement), hygiene status (limb not clean/limb clean), and acute adenolymphagitis (ADL) attack during the previous 6 months (no/yes), The effects are presented as the estimate (*β*) and the 95% CI. BSL = baseline; DOX 200 = doxycycline 200 mg; DOX 100 = doxycycline 100 mg; 6m = 6 months.

Because there is as yet no LF-specific disability assessment tool to capture the unique challenges faced by these patients, we opted to use the WHODAS 2.0 assessment to measure QoL at baseline, 12 months, and 24 months, because it has been shown to be a generally valid and sensitive tool for assessing LF-related disability.^62^^,^^63^ Although some studies have reported that severity of disease, occurrence of acute episodes, and extent of lower limb involvement appear to have an appreciable impact on the overall well-being of patients with LE, we did not find significant reductions or changes in the QoL of our participants over the observation period of this study. Rather, we found mean QoL scores that remained relatively constant throughout the study duration (Figure 6A) and with no discernible differences when analyzed by gender, age, or LE stage, as previously reported.^64^^,^^65^ However, in line with other groups’ findings, there was a positive correlation between QoL and body weight, indicating that with an increase in weight there is a decrease in the lived QoL (estimate: 0.06; *P* = 0.0232).^66^ Even more importantly, it could be seen here also that an ADL episode during the previous 6 months had a negative impact on QoL (estimate: 17.16; *P* <0.0001), as was the case if both legs were affected at baseline (estimate: 2.61, *P* = 0.004). The reasons for the lack of significant differences in QoL across various demographic features of our study population could relate to the population’s specific cultural and social factors or to the technical tools used to investigate the issues. In either case, the burden of lymphatic disease on affected people and populations is so significant that it remains incumbent on research investigators to create the tools and strategies for care that are necessary to address such challenges more successfully in the future.

### Limitations of the study.

#### Randomization.

There was no separate randomization per LE stage planned for this study, which resulted in significantly more stage 3 participants in the placebo group than in the DOX 100 group. We adjusted for this effect by using both variables (staging and treatment) in the multivariate analyses.

#### Recall bias in ADL reporting.

One of the primary limitations of our study was the reliance on patient recall to report the occurrence of ADL episodes. This introduces a potential source of recall bias, as patients may not have accurately remembered or reported all ADL episodes they experienced during the study. Objective clinical examination during ADL attacks would have provided more accurate data.

#### Suitability of WHODAS tool.

The assessment of QoL using the WHODAS tool for tracking changes in disability over time in LE patients participating in community-based surveys might not be suitable for all epidemiological settings. This tool might not fully capture the challenges faced by individuals in regions where there is stigmatization and where social support is lacking. Patients in such settings might not openly declare their inability to cope with life because of fear or social pressure.

## CONCLUSION

Our study, together with the other trials in this series, clearly shows the extent of improvement (including lack of disease progression) that can be achieved if a stringent hygiene protocol is applied to LE management. The study could also show a halt in progression by the use of DOX in addition to the stringent hygiene measures when both legs if affected at baseline were included in the analysis. In addition, the results of the study emphasize the potential of DOX as a valuable treatment option for LE to reduce ADL and highlight with the two favorable outcomes for DOX the importance of integrated care by combining it with specific hygiene measures. Addressing the challenges associated with MMDP programs through such integrated approaches is essential to improving patients’ clinical outcomes and the overall management of filarial LE.

## Supplemental Materials

10.4269/ajtmh.24-0049 Supplemental Materials

## References

[1] World Health Organization , 2021. *Global Programme to Eliminate Lymphatic Filariasis: Progress Report, 2021*. Available at: https://www.who.int/publications/i/item/who-wer9741-513-524. Accessed September 17, 2023.

[2] World Health Organization , 2023. *Lymphatic Filariasis.* Available at: https://www.who.int/news-room/fact-sheets/detail/lymphatic-filariasis. Accessed September 17, 2023.

[3] Medeiros ZM Vieira AVB Xavier AT Bezerra GSN Lopes MFC Bonfim CV Aguiar-Santos AM, 2022. Lymphatic filariasis: A systematic review on morbidity and its repercussions in countries in the Americas. Int J Environ Res Public Health 19: 316. 10.3390/ijerph19010316 PMC8751179 35010576

[4] World Health Organization , 2021. Lymphatic Filariasis: Managing Morbidity and Preventing Disability: An Aide-Mémoire for National Programme Managers, 2nd ed. Geneva, Switzerland: WHO.

[5] World Health Organization , 2020. Ending the Neglect to Attain the Sustainable Development Goals: A Road Map for Neglected Tropical Diseases 2021–2030. Geneva, Switzerland: WHO.

[6] Fitzpatrick C Engels D, 2016. Leaving no one behind: A neglected tropical disease indicator and tracers for the Sustainable Development Goals. Int Health 8 *(Suppl_1):* i15–i18. 26940304 10.1093/inthealth/ihw002 PMC4777229

[7] Horton J , 2020. The design and development of a multicentric protocol to investigate the impact of adjunctive doxycycline on the management of peripheral lymphoedema caused by lymphatic filariasis and podoconiosis. Parasit Vectors 13: 155. 32228663 10.1186/s13071-020-04024-2 PMC7106687

[8] Seim AR Dreyer G Addiss DG, 1999. Controlling morbidity and interrupting transmission: Twin pillars of lymphatic filariasis elimination. Rev Soc Bras Med Trop 32: 325–328. 10380574 10.1590/s0037-86821999000300022

[9] Tanzania Ministry of Health , 2021. *The United Republic of Tanzania Sustainability Plan for the Neglected Tropical Diseases Control Program (July 2021–June 2026)*: *Tanzania Mainland.* Available at: https://www.moh.go.tz/storage/app/uploads/public/620/b58/bc2/620b58bc2761a915449308.pdf. Accessed January 2, 2024.

[10] Mwingira U , 2017. Lymphatic filariasis patient identification in a large urban area of Tanzania: An application of a community-led mHealth system. PLoS Negl Trop Dis 11: e0005748. 28708825 10.1371/journal.pntd.0005748 PMC5529014

[11] Kalinga A , 2022. The viability of utilising phone-based text messages in data capture and reporting morbidities due to lymphatic filariasis by community health workers: A qualitative study in Kilwa district, Tanzania. BMC Health Serv Res 22: 924. 35854308 10.1186/s12913-022-08256-z PMC9295502

[12] John W Mushi V Tarimo D Mwingira U, 2021. Community participation in the mass drug administration and their knowledge, attitudes, and practices on management of filarial lymphoedema in Lindi District, Tanzania: A cross-sectional study. Afro-Egyptian J Infect Endemic Dis 11: 369–381.

[13] Mackenzie C Kapa DR Krishnasastry S Douglass J Hoerauf A Ottesen E, 2024. Managing lymphedema induced by lymphatic filariasis: Implementing and improving care at the individual and programmatic levels. Am Trop Med Hyg 111 *(Suppl 4):* 3–21. 10.4269/ajtmh.23-0905 PMC11448485 39084208

[14] Brown S Dayan JH Coriddi M Campbell A Kuonqui K Shin J Park HJ Mehrara BJ Kataru RP, 2022. Pharmacological treatment of secondary lymphedema. Front Pharmacol 13: 828513. 35145417 10.3389/fphar.2022.828513 PMC8822213

[15] Forte AJ Boczar D Huayllani MT Lu X McLaughlin SA, Pharmacotherapy agents in lymphedema treatment: A systematic review. Cureus 11: e6300. 31815082 10.7759/cureus.6300 PMC6897350

[16] Lupenza E Gasarasi DB Minzi OM, 2021. Lymphatic filariasis, infection status in *Culex* quinquefasciatus and *Anopheles* species after six rounds of mass drug administration in Masasi District, Tanzania. Infect Dis Poverty 10: 20. 33648600 10.1186/s40249-021-00808-5 PMC7919328

[17] Churko C Yohanes T Kassahun AB Desalegn N Endashaw G Asfaw MA, 2021. Foot care practice and associated factors among patients with lymphoedema in Boreda district, Gamo zone, southern Ethiopia, 2020. Implications for elimination of podoconiosis and lymphatic filariasis. J Foot Ankle Res 14: 51. 34376203 10.1186/s13047-021-00490-8 PMC8353830

[18] Debrah AY , 2006. Doxycycline reduces plasma VEGF-C/sVEGFR-3 and improves pathology in lymphatic filariasis. PLoS Pathog 2: e92. 17044733 10.1371/journal.ppat.0020092 PMC1564427

[19] Hoerauf A Volkmann L Hamelmann C Adjei O Autenrieth IB Fleischer B Büttner DW, 2000. Endosymbiotic bacteria in worms as targets for a novel chemotherapy in filariasis. Lancet 355: 1242–1243. 10770311 10.1016/S0140-6736(00)02095-X

[20] Hoerauf A Mand S Adjei O Fleischer B Büttner DW, 2001. Depletion of *Wolbachia* endobacteria in *Onchocerca volvulus* by doxycycline and microfilaridermia after ivermectin treatment. Lancet 357: 1415–1416. 11356444 10.1016/S0140-6736(00)04581-5

[21] Taylor MJ Makunde WH McGarry HF Turner JD Mand S Hoerauf A, 2005. Macrofilaricidal activity after doxycycline treatment of *Wuchereria bancrofti*: A double-blind, randomised placebo-controlled trial. Lancet 365: 2116–2121. 15964448 10.1016/S0140-6736(05)66591-9

[22] Hoerauf A , 2008. *Wolbachia* endobacteria depletion by doxycycline as antifilarial therapy has macrofilaricidal activity in onchocerciasis: A randomized placebo-controlled study. Med Microbiol Immunol (Berl) 197: 295–311. 17999080 10.1007/s00430-007-0062-1 PMC2668626

[23] Turner JD , 2010. Macrofilaricidal activity after doxycycline only treatment of *Onchocerca volvulus* in an area of Loa loa co-endemicity: A randomized controlled trial. PLoS Negl Trop Dis 4: e660. 20405054 10.1371/journal.pntd.0000660 PMC2854122

[24] Walker M Specht S Churcher TS Hoerauf A Taylor MJ Basáñez MG, 2015. Therapeutic efficacy and macrofilaricidal activity of doxycycline for the treatment of river blindness. Clin Infect Dis 60: 1199–1207. 25537873 10.1093/cid/ciu1152 PMC4370165

[25] Debrah AY , 2015. Doxycycline leads to sterility and enhanced killing of female *Onchocerca volvulus* worms in an area with persistent microfilaridermia after repeated ivermectin treatment: A randomized, placebo-controlled, double-blind trial. Clin Infect Dis 61: 517–526. 25948064 10.1093/cid/civ363 PMC4518165

[26] Mand S , 2012. Doxycycline improves filarial lymphedema independent of active filarial infection: A randomized controlled trial. Clin Infect Dis 55: 621–630. 22610930 10.1093/cid/cis486 PMC3412691

[27] Coulibaly YI , 2024. Effect of adding a six-week course of doxycycline to intensive hygiene-based care for improving lymphedema in a rural setting of mali: A double-blind, randomized controlled 24-month trial. Am J Trop Med Hyg. doi: 10.4269/ajtmh.23-0908. 10.4269/ajtmh.23-0908 PMC11448486 39013374

[28] Yahathugoda TC De Silva NL Ruben J Gunawardena S Weerasooriya MV Horton J Budge P Ottesen E Sullivan SM Stephens M Shen J Klarmann-Schulz U Hoerauf A Shott JP Mackenzie C. LEDoxy-SL: A placebo-controlled, double-blind, randomized, 24-month trial of six weeks of daily doxycycline plus hygiene-based essential care for reducing progression of filarial lymphedema in Sri Lanka. Am J Trop Med Hyg. doi: 10.4269/ajtmh.24-0050. 10.4269/ajtmh.24-0050 PMC11448493 39043165

[29] Krishnasastry S , Efficacy and safety of adding 6 weeks of doxycycline to the essential package of care to treat filarial lymphedema – a double blind, randomized controlled trial in Southern India. Am Trop Med Hyg 111 *(Suppl 4):* 83–93. 10.4269/ajtmh.24-0337 PMC11448484 39362214

[30] The United Republic of Tanzania, Ministry of Finance and Planning , 2022. *The* 2022 *Population and Housing Census: Administrative Units Population Distribution Report*. Available at: https://www.nbs.go.tz/nbs/takwimu/Census2022/Administrative_units_Population_Distribution_Report_Tanzania_volume1a.pdf. Accessed October 11, 2023.

[31] Dreyer G Addiss D Bettinger J Dreyer P Noröes J Ray F, 2001. *Lymphoedema Staff Manual: Treatment and Prevention of Problems Associated with Lymphatic Filariasis.* Available at: https://iris.who.int/bitstream/handle/10665/67224/WHO_CDS_CPE_CEE_2001.26a.pdf?sequence=1.

[32] Dreyer G, 2019. *New Hope for People with Lymphoedema*. Available at: https://www.gaelf.org/new-hope-for-people-with-lymphedema-produced-by-cdc. Accessed October 1, 2023.

[33] World Health Organization , 2010. *Measuring Health and Disability: Manual for WHO Disability Assessment Schedule WHODAS 2.0.* Available at: https://iris.who.int/handle/10665/43974. Accessed July 30, 2024.

[34] Zhou C Yahathugoda C De Silva L Rathnapala U Owen G Weerasooriya M Rao RU Weil GJ Budge PJ, 2019. Portable infrared imaging for longitudinal limb volume monitoring in patients with lymphatic filariasis. PLoS Negl Trop Dis 13: e0007762. 31584959 10.1371/journal.pntd.0007762 PMC6795459

[35] Yahathugoda C Weiler MJ Rao R De Silva L Dixon JB Weerasooriya MV Weil GJ Budge PJ, 2017. Use of a novel portable three-dimensional imaging system to measure limb volume and circumference in patients with filarial lymphedema. Am J Trop Med Hyg 97: 1836–1842. 29141750 10.4269/ajtmh.17-0504 PMC5805069

[36] McMahon JE Marshall TFdC Vaughan JP Abaru DE, 1979. Bancroftian filariasis: A comparison of microfilariae counting techniques using counting chamber, standard slide and membrane (Nuclepore) filtration. Ann Trop Med Parasitol 73: 457–464. 393190 10.1080/00034983.1979.11687285

[37] Harris PA Taylor R Thielke R Payne J Gonzalez N Conde JG, 2009. Research electronic data capture (REDCap)—A metadata-driven methodology and workflow process for providing translational research informatics support. J Biomed Inform 42: 377–381. 18929686 10.1016/j.jbi.2008.08.010 PMC2700030

[38] Harris PA , 2019. The REDCap consortium: Building an international community of software platform partners. J Biomed Inform 95: 103208. 31078660 10.1016/j.jbi.2019.103208 PMC7254481

[39] Andersen PK Gill RD, 1982. Cox’s regression model for counting processes: A large sample study. Ann Stat 10: 1100–1120.

[40] Ullah S Gabbett TJ Finch CF, 2014. Statistical modelling for recurrent events: An application to sports injuries. Br J Sports Med 48: 1287–1293. 22872683 10.1136/bjsports-2011-090803 PMC4145455

[41] Hoerauf A, 2008. Filariasis: New drugs and new opportunities for lymphatic filariasis and onchocerciasis. Curr Opin Infect Dis 21: 673–681. 18978537 10.1097/QCO.0b013e328315cde7

[42] Taylor MJ Hoerauf A Bockarie M, 2010. Lymphatic filariasis and onchocerciasis. Lancet 376: 1175–1185. 20739055 10.1016/S0140-6736(10)60586-7

[43] Makunde W Kamugisha L Makunde R Malecela M Kitua A, 2009. Hospital-based safety and tolerability study to assess efficacy of oral doxycycline in the treatment of *Wuchereria bancrofti* infection in north-eastern Tanzania. Tanzan J Health Res 8: 128–133. 10.4314/thrb.v8i3.45109 18254502

[44] Tamarozzi F Tendongfor N Enyong PA Esum M Faragher B Wanji S Taylor MJ, 2012. Long term impact of large scale community-directed delivery of doxycycline for the treatment of onchocerciasis. Parasit Vectors 5: 53. 22433114 10.1186/1756-3305-5-53 PMC3350421

[45] Turner JD Mand S Debrah AY Muehlfeld J Pfarr K McGarry HF Adjei O Taylor MJ Hoerauf A, 2006. A randomized, double-blind clinical trial of a 3-week course of doxycycline plus albendazole and ivermectin for the treatment of *Wuchereria bancrofti* infection. Clin Infect Dis 42: 1081–1089. 16575724 10.1086/501351

[46] Hoerauf A , 2009. Efficacy of 5-week doxycycline treatment on adult *Onchocerca volvulus* . Parasitol Res 104: 437–447. 18850111 10.1007/s00436-008-1217-8

[47] Debrah AY Mand S Marfo-Debrekyei Y Batsa L Albers A Specht S Klarmann U Pfarr K Adjei O Hoerauf A, 2011. Macrofilaricidal activity in *Wuchereria bancrofti* after 2 weeks treatment with a combination of rifampicin plus doxycycline. J Parasitol Res 2011: 201617. 21687646 10.1155/2011/201617 PMC3112504

[48] Debrah LB , 2024. Adherence to hygiene protocols and doxycycline therapy in ameliorating lymphatic filariasis morbidity in an endemic area post-interruption of disease transmission in Ghana. Am Trop Med Hyg 111 *(Suppl 4):* 66–82. 10.4269/ajtmh.24-0313 PMC11448491 39362213

[49] Vaqas B Ryan TJ, 2003. Lymphoedema: Pathophysiology and management in resource-poor settings—Relevance for lymphatic filariasis control programmes. Filaria J 2: 4. 12685942 10.1186/1475-2883-2-4 PMC153482

[50] Dellar R Ali O Kinfe M Mengiste A Davey G Bremner S Semrau M Fekadu A, 2022. Effect of a community-based holistic care package on physical and psychosocial outcomes in people with lower limb disorder caused by lymphatic filariasis, podoconiosis, and leprosy in Ethiopia: Results from the EnDPoINT Pilot Cohort Study. Am J Trop Med Hyg 107: 624–631. 35895351 10.4269/ajtmh.21-1180 PMC9490655

[51] Martindale S Mkwanda SZ Smith E Molyneux D Stanton MC Kelly-Hope LA, 2014. Quantifying the physical and socio-economic burden of filarial lymphoedema in Chikwawa District, Malawi. Trans R Soc Trop Med Hyg 108: 759–767. 25270880 10.1093/trstmh/tru154

[52] Barrett C Chiphwanya J Chaponda L Matipula DE Turner JD Taylor MJ Read JM Kelly-Hope LA, 2023. Mental health conditions in people affected by filarial lymphoedema in Malawi: Prevalence, associated risk factors and the impact of an enhanced self-care intervention. Int Health 15 *(* Suppl 3 *):* iii14–iii27. 38118160 10.1093/inthealth/ihad064 PMC10732670

[53] Ali O Kinfe M Semrau M Tora A Tesfaye A Mengiste A Davey G Fekadu A, 2021. A qualitative study on the implementation of a holistic care package for control and management of lymphoedema: Experience from a pilot intervention in northern Ethiopia. BMC Health Serv Res 21: 1065. 34625080 10.1186/s12913-021-07088-7 PMC8501530

[54] Stocks ME Freeman MC Addiss DG, 2015. The effect of hygiene-based lymphedema management in lymphatic filariasis-endemic areas: A systematic review and meta-analysis. PLoS Negl Trop Dis 9: e0004171. 26496129 10.1371/journal.pntd.0004171 PMC4619803

[55] Douglass J , 2020. Addition of lymphatic stimulating self-care practices reduces acute attacks among people affected by moderate and severe lower-limb lymphedema in Ethiopia, a cluster randomized controlled trial. J Clin Med 9: 4077. 33348721 10.3390/jcm9124077 PMC7766500

[56] Addiss DG Louis-Charles J Roberts J Leconte F Wendt JM Milord MD Lammie PJ Dreyer G, 2010. Feasibility and effectiveness of basic lymphedema management in Leogane, Haiti, an area endemic for bancroftian filariasis. PLoS Negl Trop Dis 4: e668. 20422031 10.1371/journal.pntd.0000668 PMC2857874

[57] Mathiarasan L Das L Krishnakumari A, 2021. Assessment of the impact of morbidity management and disability prevention for lymphatic filariasis on the disease burden in Villupuram district of Tamil Nadu, India. Indian J Community Med 46: 657. 35068729 10.4103/ijcm.IJCM_12_21 PMC8729271

[58] De Britto RLJ Vijayalakshmi G Boopathi K Kamaraj P Supriya VK Yuvaraj J, 2020. Does the morbidity management and disability prevention (MMDP) clinic serve the filarial lymphedema (FLE) patients’ preeminent expectation? Trop Biomed 37: 66–74. 33612719

[59] Shenoy RK, 2002. Management of disability in lymphatic filariasis—An update. J Commun Dis 34: 1–14. 12718336

[60] El-Nahas H El-Shazly A Abulhassan M Nabih N Mousa N, 2011. Impact of basic lymphedema management and antifilarial treatment on acute dermatolymphangioadenitis episodes and filarial antigenaemia. J Glob Infect Dis 3: 227. 21887053 10.4103/0974-777X.83527 PMC3162808

[61] Gasarasi DB Premji ZG Mujinja PGM Mpembeni R, 2000. Acute adenolymphangitis due to bancroftian filariasis in Rufiji district, south east Tanzania. Acta Trop 75: 19–28. 10708003 10.1016/s0001-706x(99)00090-x

[62] Thomas C Narahari SR Bose KS Vivekananda K Nwe S West DP Kwasny M Kundu RV, 2014. Comparison of three quality of life instruments in lymphatic filariasis: DLQI, WHODAS 2.0, and LFSQQ. PLoS Negl Trop Dis 8: e2716. 24587467 10.1371/journal.pntd.0002716 PMC3930502

[63] Budge PJ Little KM Mues KE Kennedy ED Prakash A Rout J Fox LM, 2013. Impact of community-based lymphedema management on perceived disability among patients with lymphatic filariasis in Orissa State, India. PLoS Negl Trop Dis 7: e2100. 23516648 10.1371/journal.pntd.0002100 PMC3597476

[64] Babu BV Nayak AN Rath K Kerketta AS, 2006. Use of the Dermatology Life Quality Index in filarial lymphoedema patients. Trans R Soc Trop Med Hyg 100: 258–263. 16289632 10.1016/j.trstmh.2005.05.022

[65] Harichandrakumar KT Krishnamoorthy K Kumari AK Das LK, 2006. Health status of lymphatic filariasis assessed from patients using seven domains five levels (7D5L) instrument. Acta Trop 99: 137–143. 17026947 10.1016/j.actatropica.2006.07.009

[66] Singh RP De Britto L Vijayalakshmi G, 2020. A study on “clinical epidemiology of filarial lymphedema patients attending filariasis clinic in Pondicherry”. Clin Epidemiol Glob Health 8: 915–919.

