# Supplementary material for: Efficacy of Intensified Hygiene Measures with or without the Addition of Doxycycline in the Management of Filarial Lymphedema: A Randomized Double-Blind, Placebo-Controlled Clinical Trial in Tanzania
Source: Am J Trop Med Hyg. 2024 Aug 27;111(4 Suppl):33–51. doi: 10.4269/ajtmh.24-0049 (PMC11448492; doi:10.4269/ajtmh.24-0049)
Supplement: Supplemental Materials [file tpmd240049.SD1.pdf]

Supplementary figure 1 Population Distribution by Council, Pwani Region; 2022

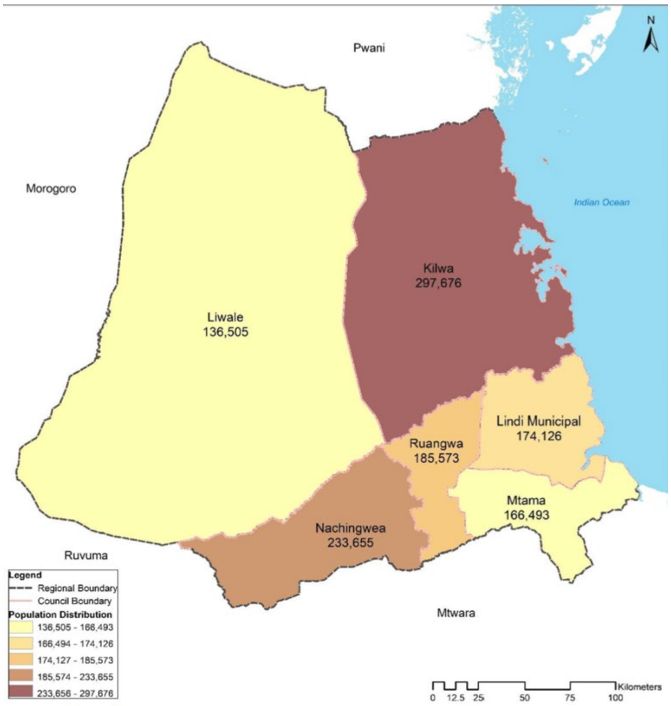

Supplementary figure 2 Population Distribution by Council, Pwani Region; 2022 PHC

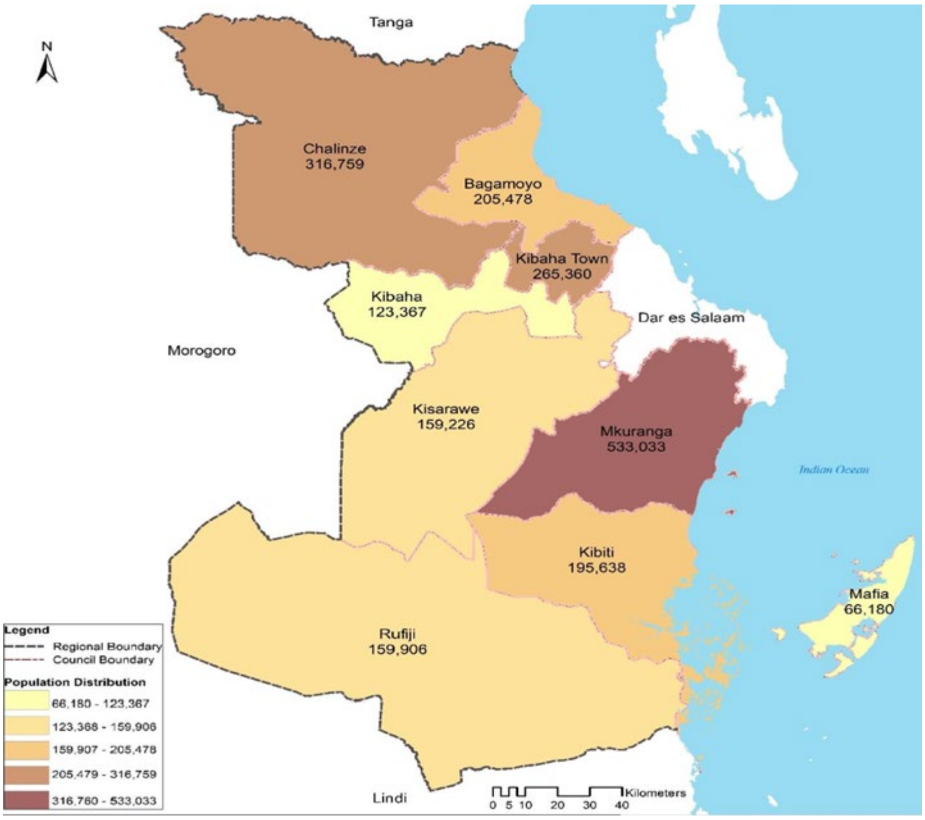

Supplementary figure 3 Stage progression and stage improvement (PP collective)

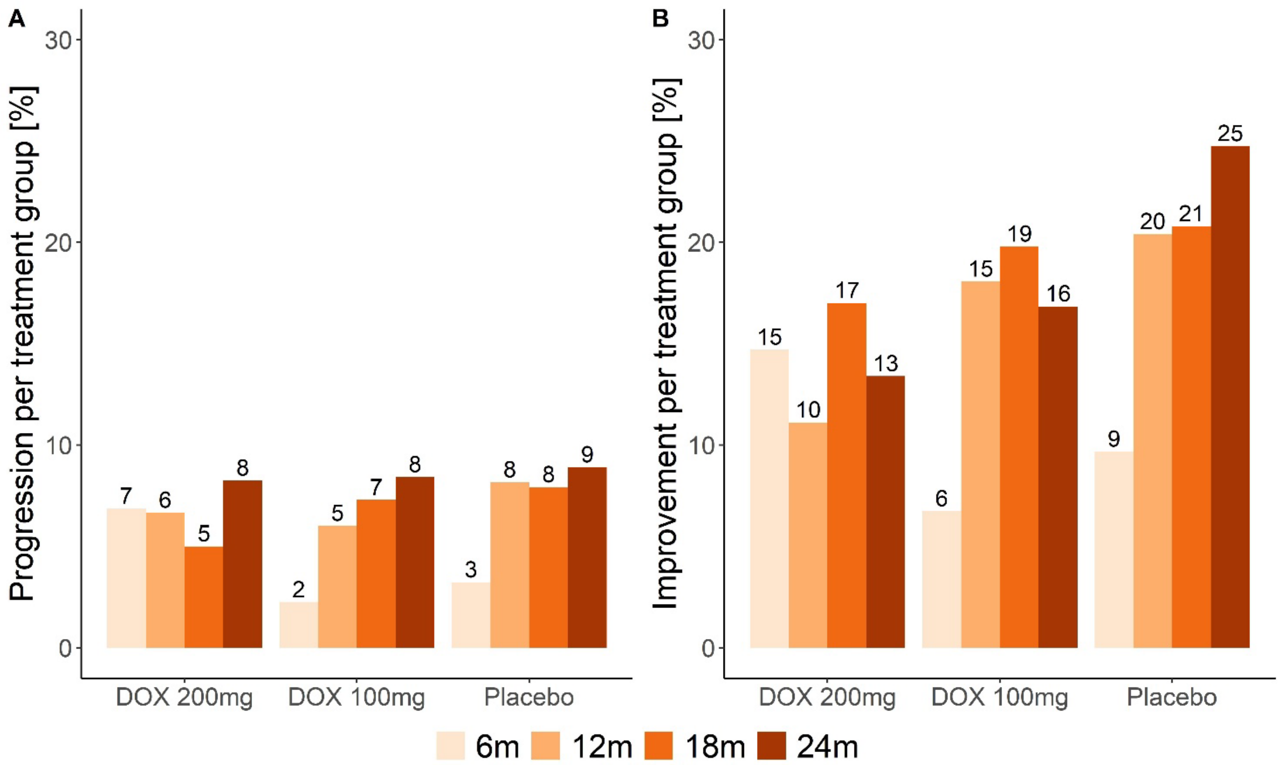

In the left diagram (A) the percentage as well as the number of participants who had a (worsening) of their LE is shown per follow-up and for each treatment separately; in the right diagram (B) the same is shown for *improvement* of LE. The diagrams represent the data of the PP collective. The percentages are always calculated to the total number participants who were present at the particular follow-up and followed treatment per protocol and can therefore vary among the time points.

Supplementary figure 4a Sankey diagram (for all legs affected at baseline)

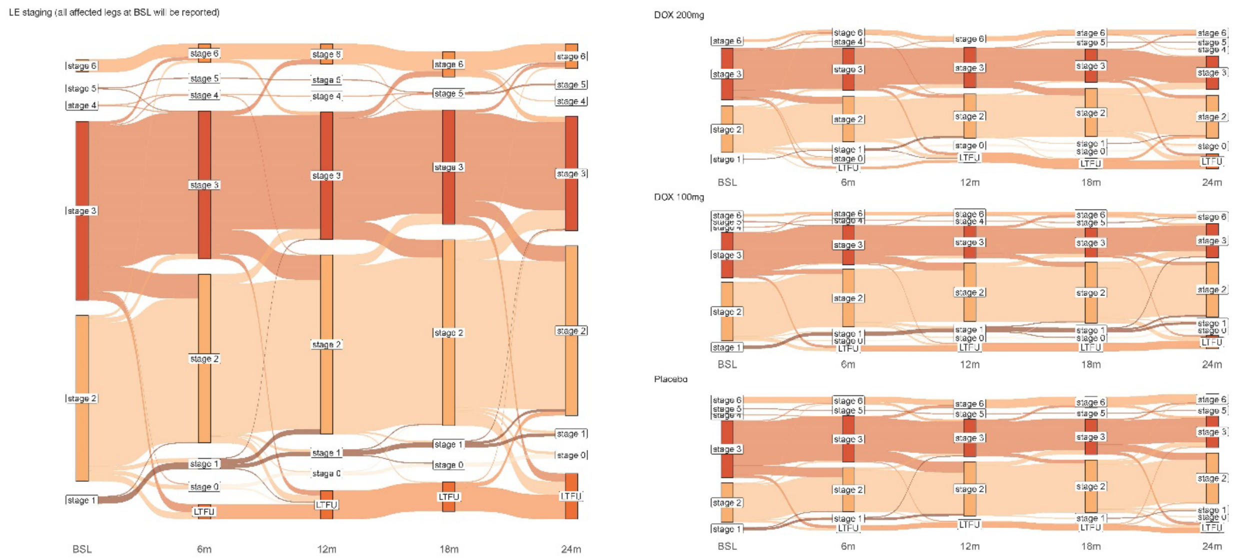

The Sankey-diagram depicts the stage changes over the whole treatment period. On the left side it is shown for all groups together, on the right side separately for each treatment arm. The diagram represents the data of the ITT collective.

Supplementary figure 4b Forest plot – multivariable analysis for stage improvement over time (for all legs affected at baseline)

		OR (95% CI)
sex	female	1.00 (0.73−1.36)
	male	reference
age at BSL	per year	0.99 (0.97−1.01)
weight at BSL	per kg	1.00 (0.99−1.01)
endemic	per year	1.00 (0.99−1.01)
treatment	DOX 200mg	0.66 (0.46−0.93)
	DOX 100mg	0.90 (0.64−1.27)
	Placebo	reference
LE−stage	stage >3	0.51 (0.27−0.95)
	stage 1+2	0.15 (0.10−0.22)
	stage 3	reference
Hygiene	limb not clean	0.83 (0.37−1.84)
	limb clean	reference
ADL during 6m	no	1.42 (0.91−2.22)
	yes	reference
Region	Pwani	0.56 (0.38−0.84)
	Lindi	reference
Rain during 6m	yes	0.92 (0.67−1.26)
	no	reference

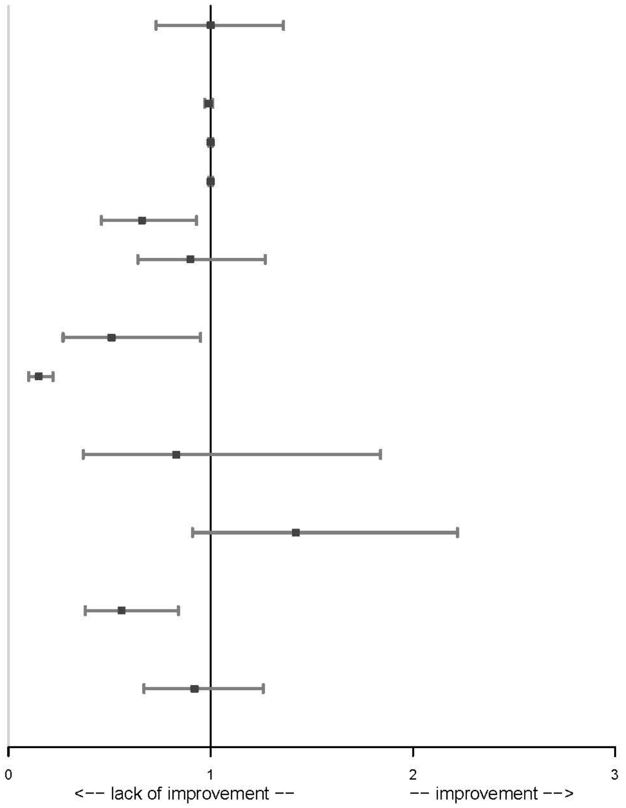

The Forest plot depicts the different co-variables that were used in a multivariable logistic regression model (Proc GENMOD, SAS) for the time-dependent outcome variable “improvement”. The following baseline co-variables were used for this model sex (male/female), age, weight, years in endemic area, LE staging (stage 1 or 2/stage 3), treatment (DOX 200/DOX 100/Placebo), region (Pwani/Lindi) as well as the following time-dependent co-variables (taking changes during the follow-up period into account): hygiene status (limb not clean/limb clean), ADL attack during the previous 6 months (no/yes), more days of rainy season during the previous 6 months (yes/no). Effects are presented as odds ratios (OR) with 95% confidence intervals (CI).

Supplementary table 1a Additional baseline data – demographics

			DOX 200mg	DOX 100mg	Placebo	Total	*p-value*
**Marital Status**	Missings		3	3	0	6	
	Never married	N (%)	6 (5%)	7 (6%)	6 (5%)	19 (5.3%)	
	Currently married	N (%)	78 (65.5%)	75 (64.7%)	75 (62%)	228 (64%)	
	Separated	N (%)	8 (6.7%)	3 (2.6%)	8 (6.6%)	19 (5.3%)	
	Divorced	N (%)	10 (8.4%)	11 (9.5%)	16 (13.2%)	37 (10.4%)	
	Widowed	N (%)	15 (12.6%)	15 (12.9%)	16 (13.2%)	46 (12.9%)	
	Cohabiting	N (%)	2 (1.7%)	5 (4.3%)	0 (0%)	7 (2%)	*0.4499*[c]
**Married**	no	N (%)	44 (36.1%)	44 (37%)	46 (38%)	134 (37%)	
	yes	N (%)	78 (63.9%)	75 (63%)	75 (62%)	228 (63%)	*0.9432*[a]
**Main work status**	Paid work	N (%)	0 (0%)	2 (1.7%)	3 (2.5%)	5 (1.4%)	
	Self-employed	N (%)	114 (95.8%)	107 (92.2%)	113 (93.4%)	334 (93.8%)	
	Non-paid work	N (%)	0 (0%)	1 (0.9%)	1 (0.8%)	2 (0.6%)	
	Student	N (%)	1 (0.8%)	0 (0%)	1 (0.8%)	2 (0.6%)	
	Keeping house/ homemaker	N (%)	0 (0%)	1 (0.9%)	1 (0.8%)	2 (0.6%)	
	Retired	N (%)	1 (0.8%)	0 (0%)	1 (0.8%)	2 (0.6%)	
	Unemployed (health reason)	N (%)	2 (1.7%)	3 (2.6%)	1 (0.8%)	6 (1.7%)	
	Unemployed (other reasons)	N (%)	1 (0.8%)	1 (0.9%)	0 (0%)	2 (0.6%)	
	Other	N (%)	0 (0%)	1 (0.9%)	0 (0%)	1 (0.3%)	*0.8116*[c]

[a] Fishers exact test [b] ANOVA [c] Chi-square test [d] Kruskal-Wallis test

Supplementary table 1b Additional baseline data – LE characteristics

			DOX 200mg	DOX 100mg	Placebo	Total	*p-value*
**LE staging right leg**	0 - No abnormality	N (%)	35 (28.7%)	58 (48.7%)	56 (46.3%)	149 (41.2%)	
	1 - Swelling is reversible	N (%)	1 (0.8%)	3 (2.5%)	3 (2.5%)	7 (1.9%)	
	2 - Swelling is not reversible	N (%)	42 (34.4%)	31 (26.1%)	28 (23.1%)	101 (27.9%)	
	3 - Presence of shallow skin	N (%)	43 (35.2%)	23 (19.3%)	28 (23.1%)	94 (26%)	
	4 - Presence of skin knobs	N (%)	0 (0%)	1 (0.8%)	1 (0.8%)	2 (0.6%)	
	5 - Presence of deep skin folds	N (%)	0(0%)	1 (0.8%)	0(0%)	1 (0.3%)	
	6 - Presence of mossy lesions	N (%)	1 (0.8%)	2 (1.7%)	5 (4.1%)	8 (2.2%)	*n.d.*
**LE staging Left leg**	0 - No abnormality	N (%)	48 (39.3%)	38 (31.9%)	36 (29.8%)	122 (33.7%)	
	1 - Swelling is reversible	N (%)	0 (0%)	1 (0.8%)	1 (0.8%)	2 (0.6%)	
	2 - Swelling is not reversible	N (%)	32 (26.2%)	44 (37%)	27 (22.3%)	103 (28.5%)	
	3 - Presence of shallow skin	N (%)	39 (32%)	35 (29.4%)	52 (43%)	126 (34.8%)	
	5 - Presence of deep skin folds	N (%)	0 (0%)	0 (0%)	1 (0.8%)	1 (0.3%)	
	6 - Presence of mossy lesions	N (%)	3 (2.5%)	1 (0.8%)	4 (3.3%)	8 (2.2%)	*n.d.*

[a] Fishers exact test [b] ANOVA [c] Kruskal-Wallis test

Supplementary table 1c Additional baseline data – ADL history

		DOX 200mg	DOX 100mg	Placebo	*Total*	*p-value*
**Number of attacks within the last year (all patients)**	N	119	110	111	340	
	median, iqr	1; 2	1; 2	1; 2	1; 2	
	95% CI median	[1; 2]	[1; 1]	[1; 1]	[1; 1]	
	min - max	0-4	0-5	0-6	0-6	*0.4476*[c]
**Duration of attacks (average within the last year (days)) (all patients)**	N	122	117	120	359	
	median, iqr	3; 4	3; 3	3; 4	3; 4	
	95% CI median	[3; 3]	[2; 3]	[1; 3]	[3; 3]	
	min - max	0-30	0-14	0-30	0-30	*0.3549*[c]
**Duration of attacks (average within the last year (days)) (only patients who had attacks)**	N	82	76	74	232	
	median, iqr	3; 2	3; 1	4; 2	3; 1	
	95% CI median	[3; 4]	[3; 3]	[3; 4]	[3; 4]	
	min - max	1-30	1-14	1-30	1-30	***0.013***[c]

[a] Fishers exact test [b] ANOVA [c] Kruskal-Wallis test [d] Chi-square test

Supplementary table 1d Additional baseline data – Hygiene assessment

		DOX 200mg	DOX 100mg	Placebo	Total	*p-value*
**Observer Assessed Cleanliness Score**	N	122	119	121	362	
	median, iqr	1; 0	1; 0	1; 0	1; 0	
	95% CI median	[1; 1]	[1; 1]	[1; 1]	[1; 1]	
	min - max	0.7-1	0.3-1	0.6-1	0.3-1	0.3892[c]
**Observer Assessed Skin Score**	N	122	119	121	362	
	median, iqr	1; 0	1; 0.1	1; 0	1; 0	
	95% CI median	[1; 1]	[1; 1]	[1; 1]	[1; 1]	
	min - max	0.7-1	0.8-1	0.6-1	0.6-1	*0.5871*[c]
**Self Assessed Hygiene Score**	N	27	30	28	85	
	median, iqr	0.6; 0.1	0.6; 0.1	0.7; 0	0.6; 0	
	95% CI median	[0.6; 0.7]	[0.6; 0.6]	[0.6; 0.7]	[0.6; 0.7]	
	min - max	0.5-0.9	0.4-0.9	0.5-0.8	0.4-0.9	*0.201*[c]

[a] Fishers exact test [b] ANOVA [c] Kruskal-Wallis test

Supplementary table 1e Additional baseline data – WHODAS 2.0

		DOX 200mg	DOX 100mg	Placebo	Total	*p-value*
**WHODAS 2.0 Physical LF Sub-score (Items S1, S7-S9)**	N	119	116	121	356	
	median, iqr	0; 2.1	0; 2.1	0; 2.1	0; 2.1	
	95% CI median	[0; 0]	[0; 0]	[0; 0]	[0; 0]	
	min - max	0-18.8	0-22.9	0-33.3	0-33.3	*0.7496*[c]
**WHODAS 2.0 Not Physical LF Sub-Score (Items S2-S6, S10-S12)**	N	119	116	121	356	
	median, iqr	0; 2.1	0; 0	0; 2.1	0; 2.1	
	95% CI median	[0; 0]	[0; 0]	[0; 0]	[0; 0]	
	min - max	0-27.1	0-31.3	0-47.9	0-47.9	*0.1282*[c]
**H1: Overall, in the past 30 days, how many days were these difficulties present?**	N	119	116	121	356	
	median, iqr	0; 2	0; 2	0; 2	0; 2	
	95% CI median	[0; 0]	[0; 0]	[0; 0]	[0; 0]	
	min - max	0-14	0-4	0-14	0-14	*0.47*[c]
**H2: In the past 30 days, for how many days were you totally unable to carry out your usual activities or work because of any health condition?**	N	119	116	121	356	
	median, iqr	0; 0	0; 0	0; 0	0; 0	
	95% CI median	[0; 0]	[0; 0]	[0; 0]	[0; 0]	
	min - max	0-14	0-4	0-7	0-14	*0.1669*[c]
**H3: In the past 30 days, not counting the days that you were totally unable, for how many days did you cut back or reduce your usual activities or work because of any health condition?**	N	119	116	121	356	
	median, iqr	0; 0	0; 0	0; 0	0; 0	
	95% CI median	[0; 0]	[0; 0]	[0; 0]	[0; 0]	
	min - max	0-9	0-4	0-14	0-14	*0.2555*[c]

Supplementary table 2 Most reported adverse events in the period between treatment end and the 4 months follow-up

Adverse event	DOX 200mg	DOX 100mg	Placebo	Total
**Headache**	5 (50%)	2 (20%)	3 (30%)	10
**Malaria**	5 (62.5%)	1 (12.5%)	2 (25%)	8
**Pyrexia**	1 (14.3%)	3 (42.9%)	3 (42.9%)	7
**Lymphangitis**	3 (50%)	0 (0%)	3 (50%)	6
**Injury**	0 (0%)	3 (60%)	2 (40%)	5
**Wound**	0 (0%)	1 (33.3%)	2 (66.7%)	3
**Other***	9 (42.9%)	6 (28.6%)	6 (28.6%)	21
**Total**	**23 (38.3%)**	**16 (26.7%)**	**21 (35%)**	**60**

* Other – occurrence < 3 (i.e. arthralgia, caries, dizziness, myalgia, vomiting, stroke, chest pain, cough, diarrhoea, dyspnea, malaise, pain in extremity, peripheral swelling, pyelonephritis, rhinorrhoea, scratch)

Supplementary table 3 FTS and MF data

	Filarial test strip (FTS)		Baseline	6 months	12 months	24 months
**DOX 200mg**	positive	N (%)	1 (0.8%)	0 (0%)	0 (0%)	0 (0%)
	negative	N (%)	121 (99.2%)	91 (100%)	81 (100%)	85 (100%)
**DOX 100mg**	positive	N (%)	1 (0.8%)	0 (0%)	3 (3.7%)	1 (1.2%)
	negative	N (%)	118 (99.2%)	87 (100%)	78 (96.3%)	84 (98.8%)
**Placebo**	positive	N (%)	1 (0.8%)	0 (0%)	2 (2.2%)	2 (2.3%)
	negative	N (%)	120 (99.2%)	88 (100%)	88 (97.8%)	86 (97.7%)
**Total**	positive	N (%)	3 (0.8%)	0 (0%)	5 (1.9%)	3 (1.2%)
	negative	N (%)	359 (99.2%)	266 (100%)	252 (98.1%)	255 (98.8%)
	**Microfilariae (MF)**					
**DOX 200mg**	positive	N (%)	1 (100%)	-	-	-
	negative	N (%)	0 (0%)	-	-	-
**DOX 100mg**	positive	N (%)	0 (0%)	-	0 (0%)	-
	negative	N (%)	1 (100%)	-	1 (100%)	-
	missing	N (%)	-	-	2	1
**Placebo**	positive	N (%)	0 (0%)	-	0 (0%)	1 (100%)
	negative	N (%)	1 (100%)	-	1 (100%)	0 (0%)
	missing	N (%)	-	-	1	1
**Total**	positive	N (%)	1 (33.3%)	-	0 (0%)	1 (100%)
	negative	N (%)	2 (66.7%)	-	2 (100%)	0 (0%)
	missing	N (%)	-	-	3	2

In total 11 participants were FTS positive at baseline or became positive during one of the follow-ups. Two out of the 3 participants who were FTS positive at baseline (Placebo, DOX 100mg) were already FTS negative 6 months later. The DOX 200 participant who was also the only MF positive person at baseline did not show up for the 6 months follow-up, but tested FTS negative at 12 months. There were no other participants from the DOX 200 group who became positive after treatment. However, three participants from the DOX 100 and 2 from the placebo group were tested FTS positive at 12 months. Unfortunately, for logistical reasons it was not possible to get night blood from all 5 participants but only from two, who were both MF negative. The three DOX 100 participants were tested FTS-negative again after 24 months. There are no 24 months data for the two placebo participants. The three participants (placebo N = 2, DOX 100 N = 1) who were tested positive for FTS at 24 months were all FTS negative before. In two participants it was again not possible to get night blood for logistical reasons, but the one placebo participant who was tested for MF showed a positive result.
